# Cryogenically Flexible Phosphorescent Organic Crystals that Transmit Self-Sustained Persistent Luminescence with Spatiotemporal Control

**DOI:** 10.1021/jacs.5c05733

**Published:** 2025-06-16

**Authors:** Xuesong Yang, Mingqi Zhang, Baolei Tang, Lijie Wang, Bing Yang, Liang Li, Panče Naumov, Hongyu Zhang

**Affiliations:** † State Key Laboratory of Supramolecular Structure and Materials, College of Chemistry, 12510 Jilin University, Changchun 130012, P. R. China; ‡ College of Electronic Science and Engineering, Jilin University, Changchun 130012, China; § SAFIR Novel Materials Development Lab, Sorbonne University Abu Dhabi, Abu Dhabi, P.O. Box 38044, UAE; ∥ Smart Materials Lab, 167632 New York University Abu Dhabi, Abu Dhabi, P.O. Box 129188, UAE; ⊥ Center for Smart Engineering Materials, New York University Abu Dhabi, Abu Dhabi, P.O. Box 129188, UAE; # Research Center for Environment and Materials, Macedonian Academy of Sciences and Arts, Bul. Krste Misirkov 2, Skopje MK-1000, Macedonia; ¶ Molecular Design Institute, Department of Chemistry, 5894 New York University, 100 Washington Square East, New York 10003, New York, United States

## Abstract

Concomitant long-lived phosphorescence and cryogenic elasticity in soft matter is an immensely challenging endeavor due to the contrasting effect of low temperatures on these properties. While the low temperature normally extends and enhances phosphorescence, it typically compromises mechanical elasticity by freezing the molecular motion, inevitably leading to brittleness and cracking of soft materials. In this work, we posit that the emerging class of organic crystals can overcome this intrinsic disparity and describe an organic crystalline material that meets both requirements—an exceptional elasticity of its crystals at 77 K and ultralong afterglow of up to about 30 s, the longest lifetime of a flexible organic crystal reported to date. The material, triphenylene, was prepared as elastic crystals, where the molecular rigidity and dense packing enable reversible lattice deformation and mechanical robustness on cooling, while they also result in prolonged phosphorescence at low temperatures. Crystals of this material act as dynamic phosphorescent waveguides, with their emission persisting in low temperatures and dark, demonstrating both sustained signal transmission capabilities and a unique opportunity for spatiotemporal control of the optical output. At a conceptual level, the results introduce organic crystals for time-encoded biological information transmission, providing a novel material platform for flexible, lightweight optical devices and sensors that can function in extreme environments.

## Graphical abstract

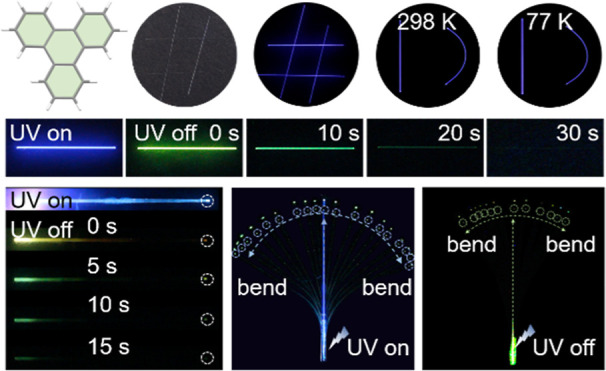

## Introduction

Flexible materials have become important components of modern technological advancements poised to overcome the inherent rigidity of silicon-based devices and potentially revolutionize several key technologies. While extensive work has been dedicated to the common soft materials such as polymers and composites, being devoid of structural defects and being structurally ordered, flexible organic crystals have only very recently stood out as a remarkable material class that combines structural adaptability with favorable optoelectronic performance.^1–8^ Their ability to withstand and absorb mechanical stress while maintaining structural integrity opens vast opportunities for applications in flexible electronics, wearable devices, and adaptive optics, which could circumvent the intrinsic disadvantage of silicon-based devices.^9–15^ The phosphorescence of some flexible organic crystals has further expanded the opportunities for flexible all-organic optics, with direct implications for sensing and anticounterfeiting technologies.^16–21^ The synergy between mechanical flexibility and persistent luminescence in these materials not only addresses the growing demand for flexible emitters, but also solves the increasing demand for durable and mechanically robust emissive materials, at a larger scale.^22–25^ A set of specific applications that require optical transduction below ambient temperatures, however, has posed a new objective for materials science research. While low temperature is known to enhance the phosphorescence of crystalline materials by thermal deactivation of possible nonradiative pathways, it usually compromises the mechanical elasticity, creating a fundamental challenge with attaining both properties simultaneously.^26–28^ At low temperatures, most soft materials become more rigid and brittle, and therefore normally flexible optoelectronics fails.^29^ Circumventing this obstacle necessitates balancing molecular packing, intermolecular interactions, and structural dynamics—factors that account for sustained integrity under extreme conditions.^30–35^ In this work, we focused on triphenylene (TPH), a classic phosphorescent emitter with long-lived phosphorescence.^36^ The tightly packed, π-stacked, rigid molecules in this solid are expected to contribute to mechanical compliance, while their rigidity and the softness of weak intermolecular interactions should aid in structural stability (Figure 1a,b). Here we report that, indeed, crystals of TPH exhibit remarkable cryogenic elasticity while maintaining both fluorescence and phosphorescence waveguiding capability, in both straight and bent configurations (Figure 1c). The material is known to be a unique phosphorescence waveguide, and the crystal flexibility makes it amenable for precise spatiotemporal control that attains dynamic afterglow propagation, even after excitation. We also demonstrate that the optical transmission of this flexible material through biological tissues is independent of the transmission path but depends on the excitation temperature, opening opportunities for bioimaging and sensing in tissues. These and other organic crystals that combine low-temperature elasticity and long-lived phosphorescence represent a groundbreaking concept with using organic crystals in cryoflexible photonics, sensing, and anticounterfeiting technologies.

**Figure 1:**
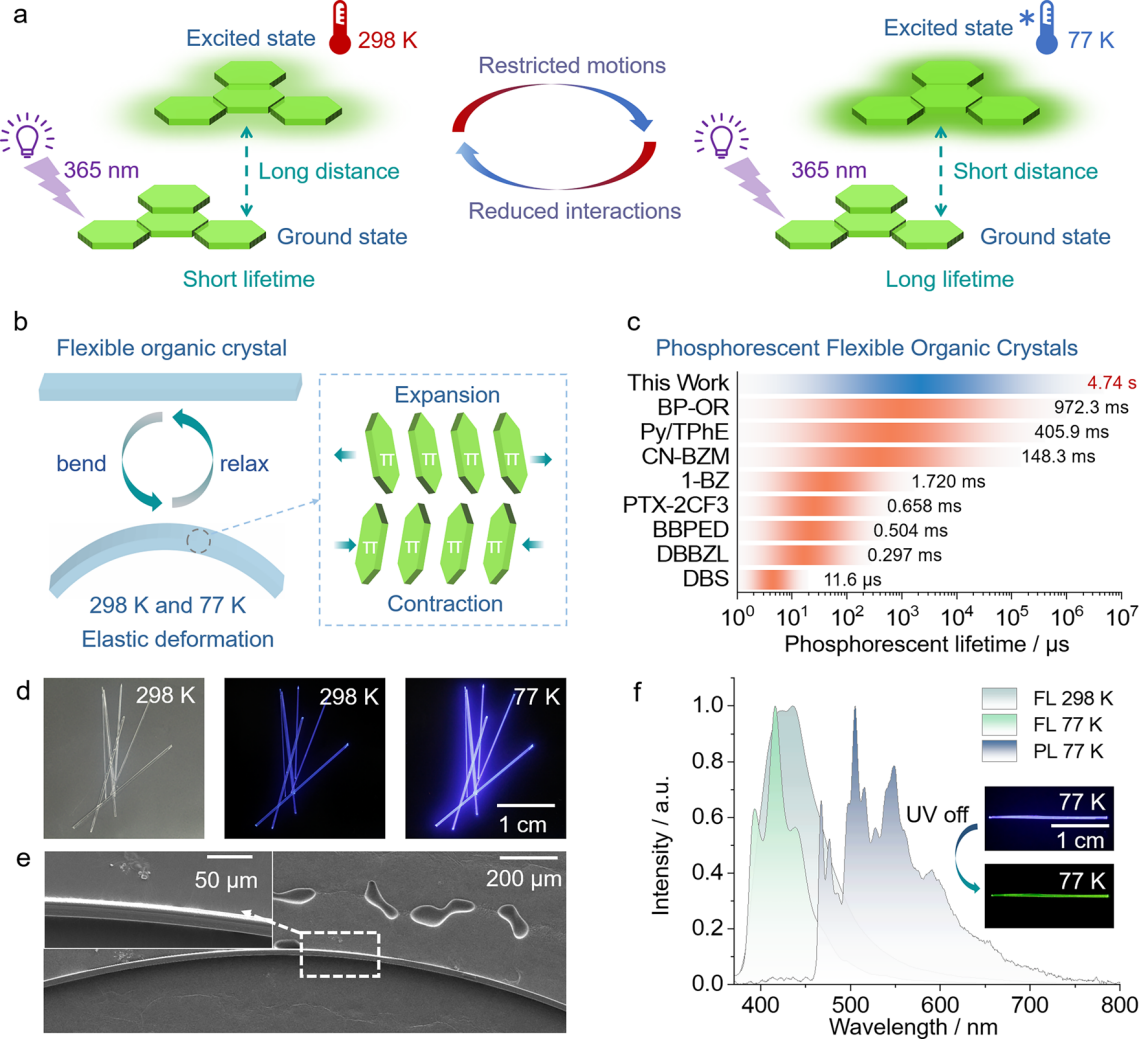
Emission properties of the flexible crystals of triphenylene (TPH). (a) Mechanism of emission from crystals at different temperatures. At room temperature, the molecules undergo thermal motion, which facilitates nonradiative transitions such as internal conversion and vibrational relaxation, thereby shortening the phosphorescence lifetime. At 77 K, the molecular motion is restricted, and the crystal structure becomes more compact and rigid, thereby effectively suppressing the nonradiative transitions and prolonging the phosphorescence lifetime. (b) Sketch of the structural changes expected to occur during the elastic bending of TPH crystals at 298 and 77 K. (c) Comparison of the phosphorescence lifetime of TPH (top blue bar) with the phosphorescence lifetime of other reported emissive flexible crystals.^16–22,25^ (d) Photographs of the TPH crystals under daylight and under UV (365 nm) radiation. (e) Scanning electron micrograph of a TPH crystal that has been bent 100 times. (f) Fluorescence (FL) and phosphorescence (PL) emission spectra recorded from TPH crystals at 298 and 77 K.

## Results and Discussion

### Preparation and Characterization of TPH Crystals

Triphenylene was purchased from a commercial supplier (Figure S1) and purified by column chromatography and vapor sublimation (Figures S2 and S3). Long, needle-like crystals that were 2–4 cm in length were obtained via solvent diffusion between dichloromethane and ethanol (v:v = 1:2, Figure 1d). The specific experimental procedure involves the addition of 3 mL of a saturated TPH dichloromethane solution into a test tube and subsequent addition (without disrupting the solvent interface) of 6 mL of ethanol. The test tube was placed at room temperature and a relative humidity of 54% and left undisturbed for 1 to 2 weeks to obtain TPH crystals (Figure S4). Although the luminescent properties of bulk TPH have been studied,^16,36^ we systematically characterized the thermal effects on the emission from the long crystals at 298 and 77 K. Upon exposure to UV light (365 nm), the crystals emit bright blue-violet fluorescence at both temperatures (Figure 1d). The crystals exhibited remarkable elasticity. As shown in Figure S5, by applying external stress to both ends of a crystal using tweezers, the crystal could be bent into a U-shape at both room temperature and low temperature without breaking. Once the external force was removed, the crystal successfully returned to its original straight shape. In addition, when one end of the crystal was fixed and the position of the other end was controlled by using a needle, the crystal could be wound into a circle (Figure S6). Scanning electron microscopy (SEM) did not show any visible damage on the crystal surface even after 100 cycles of bending and straightening which confirmed its outstanding mechanical robustness (Figure 1e). As shown in Figure 1f, the fluorescence maximum of the crystals was observed at 435 and 416 nm at 298 and 77 K, respectively, while the phosphorescence maximum was at 505 nm at 77 K. In addition, the fluorescence quantum yield of TPH crystals at 298 K, the phosphorescence quantum yield at 298 K, the fluorescence quantum yield at 77 K, and the phosphorescence quantum yield at 77 K were characterized. The results showed that the fluorescence quantum yield at 298 K, the phosphorescence quantum yield at 298 K, the fluorescence quantum yield at 77 K, and the phosphorescence quantum yield at 77 K are 55.60%, 2.89%, 58.02%, and 19.87%, respectively. Three-point bending tests were conducted on three samples to quantitatively evaluate the mechanical properties of the crystals, and linear fitting of the elastic region of the stress–strain curve (Figure S7) returned a Young’s modulus of approximately 3.51 GPa.^37,38^ To investigate the maximum force that the TPH crystals can withstand without breaking, we characterized the mechanical properties of samples with different widths and thicknesses using a three-point bending test. As shown in Figure S8, the results indicated that the maximum force that the TPH crystals can withstand was approximately 0.54 N. To quantitatively evaluate the mechanical bendability of crystals at 298 and 77 K, the maximum expansion/contraction ratio (maximum elastic strain, ε) of the inner/outer arcs of bent crystals without cracks was determined using a reported method (Figure S9).^1^ The calculated ε values for crystals were 1.48% at room temperature and 1.07% at 77 K (thickness: 0.093 mm at 298 K, and 0.067 mm at 77 K; Table S1). As shown in Table S2, the Young’s modulus of TPH is comparable to the previously reported Young’s moduli of other organic crystals.^9,29,31–33,37,39–43^

### Phosphorescent Long Afterglow of TPH Crystals

The combination of cryogenic elasticity and long-lived phosphorescence in organic crystals provides exciting opportunities for achieving multiple functionalities (Movie S1). As shown in Figure 2a, a straight TPH crystal at 77 K initially emits bright blue-violet fluorescence under 365 nm excitation. The blue fluorescence changes to vivid green phosphorescence once the UV excitation has been terminated. The green phosphorescence gradually decays over time but remains visible, even after 30 s, highlighting its exceptionally long lifetime. Remarkably, the crystals are also mechanically elastic and can be easily bent by applying force. As illustrated in Figure 2b, for a prebent crystal, the change from blue fluorescence to green phosphorescence is also observed when it is excited at 77 K. Moreover, Figure 2c shows that a straight crystal can be excited at 77 K, bent, and let recover its straight shape, and it shows the same transition in emission. Altogether, the experiments confirm both favorable mechanical flexibility and robust emissive properties of TPH crystals in a cryogenic environment. These crystals can be assembled in more elaborate emissive architectures, such as the exemplary acronyms “JLU” and “SOS”, as shown in Figure 2d,e, while they maintain their phosphorescent properties over extended time. The intensity of the emission decreases gradually over time (Figure 2f), and the phosphorescence lifetimes at 467, 505, 549, and 590 nm were determined to be 3.09 4.05, 3.01, and 3.36 s, respectively (Figure 2g). The cyclability of the long-lived phosphorescence of TPH crystals was confirmed through repeated excitations and measurement at 77 K, while the lifetime changes were monitored at 505 nm (Figures 2h and S10). To gain a deeper understanding of the phosphorescent emission mechanism of TPH crystals, we performed density functional theory (DFT) and time-dependent DFT calculations on the TPH molecule using the B3LYP/6-31G­(d) basis set.^44^ Figure S11 (Tables S3–S5) shows the absorption and emission energy levels of TPH molecules between singlet states (S_*n*_, *n* = 1–10) and triplet states (T_*n*_, *n* = 1–10), along with the corresponding computational results. Specifically, the emission energy of the S_1_ state is 3.75 eV, while the emission energy of the T_1_ state is 2.65 eV. The spin–orbit coupling (SOC) effect has been proven to be a key factor in determining whether pure organic materials can achieve phosphorescent emission. According to the Kasha’s rule, most photophysical processes occur in the lowest excited state. The calculation results (Table S6) show that the SOC value between S_1_ and T_1_ is as high as 2.74 cm^–1^, indicating that the SOC effect is significant and can effectively promote the generation of T_1_ excitons. Therefore, TPH crystals exhibit strong phosphorescence. Altogether, the experiments demonstrated the robustness of both the phosphorescence and mechanical flexibility at low temperatures. To explore whether the combination of cryogenic flexibility and persistent luminescence observed with the TPH crystals is more common and occurs in other polycyclic aromatic compounds, 15 other polycyclic aromatic compounds with different chemical structures were procured, purified using column chromatography, and crystallized. As shown in Figure S12, all but one of the polycyclic aromatic compounds do not exhibit low-temperature flexibility simultaneously with low-temperature long afterglow phosphorescence. For instance, 9,10-dibromoanthracene crystals are flexible at low temperature but are not phosphorescent, while compounds such as dibenzothiophene and benzo­[*a*]­pyrene exhibit low-temperature phosphorescence; however, they lack low-temperature flexibility. An exception was found with a derivative of triphenylene, 2,7-dibromophenanthrene, whose crystals were found to simultaneously exhibit low-temperature flexibility and low-temperature phosphorescence. This example provides an indication that this combination of properties may be found with other materials and could be a motivation for further exploration and design of polycyclic aromatic compounds with both low-temperature flexibility and phosphorescent properties.

**Figure 2:**
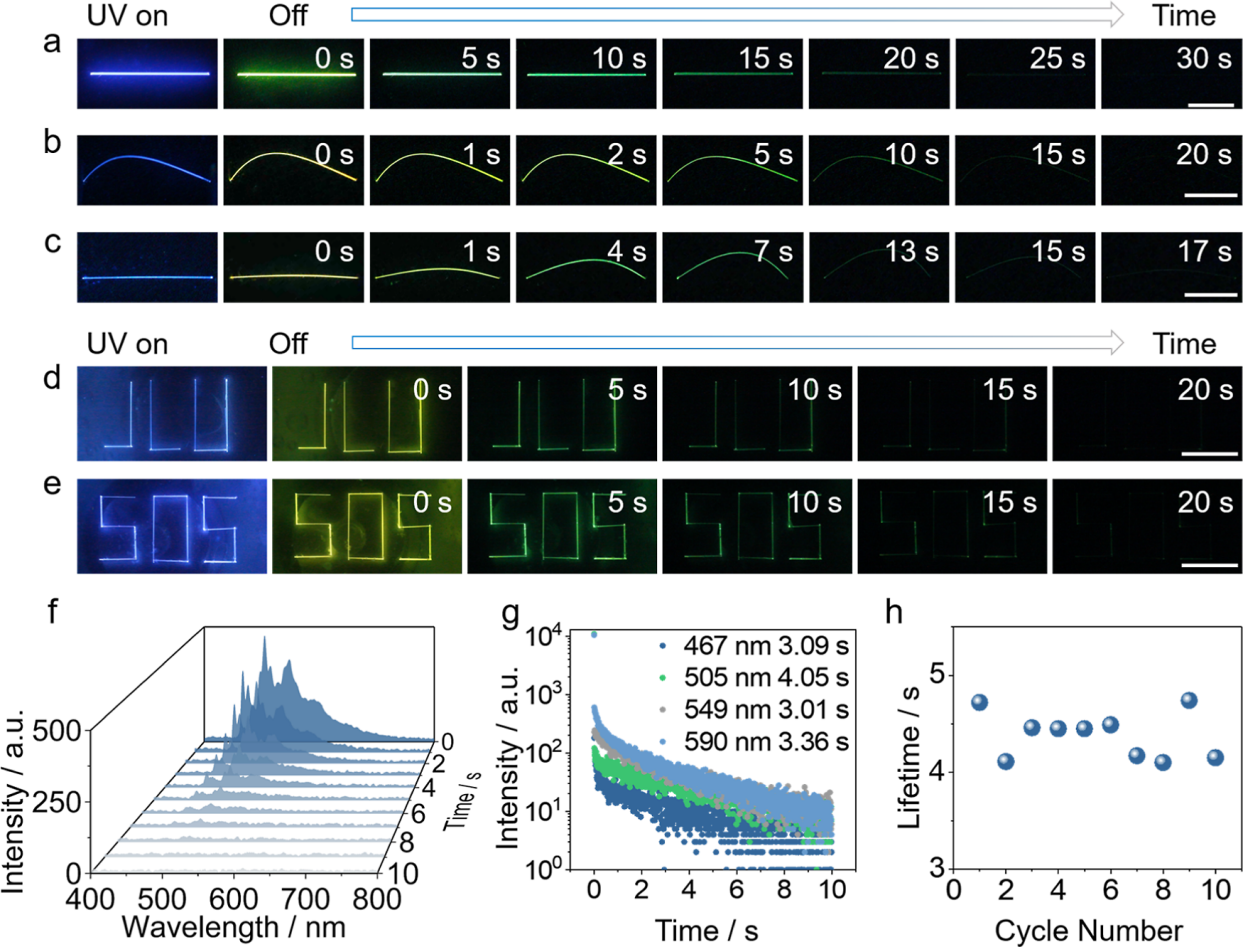
Fluorescence and phosphorescence of TPH crystals. (a–c) Time-lapse photographic sequences showing the emission after UV irradiation of a straight crystal at 77 K (a), a bent crystal at 77 K (b), a straight crystal while it was bent and let recover the straight shape at 77 K (c). (d,e) Time-lapse photographic sequence of irradiated crystals arranged in the shape of the acronyms “JLU” (d) and “SOS” (e) at 77 K. (f) Time-resolved emission spectra of TPH crystals at 77 K. (g) Decay of the emission intensity of TPH crystals recorded at varying emission wavelengths at 77 K. (h) Phosphorescence lifetimes of TPH crystals determined upon repeated excitation and measurement of the lifetime at 77 K. The length of the white scale bar in panels a–e is 5 mm.

### Structure of the TPH Crystals

To explain the elasticity of the TPH crystals, single crystal X-ray diffraction analysis was performed on a straight crystal at 298 and 100 K (Table S7). At 298 K, the crystal is orthorhombic, space group *P*2_1_2_1_2_1_, with *Z* = 4, and the unit cell parameters are *a* = 5.2752(3) Å, *b* = 13.1795(8) Å, and *c* = 16.7642(10) Å (Figure 3a). The molecules are arranged in a parallel stacking fashion, forming columnar structures via π···π interactions with an intermolecular spacing of 3.448 Å along the crystallographic [100] direction (Figure S13). Upon cooling to 100 K, the crystal symmetry is retained; however, the cell parameters change to *a* = 5.2667(3) Å, *b* = 12.8822(8) Å, and *c* = 16.6351(10) Å, corresponding to a contraction of 0.16% along the *a*-axis, 2.25% along the *b*-axis, and 0.77% along the *c*-axis (Figure 3a). Concurrently, the stacking distance of the molecules decreases slightly, from 3.448 to 3.387 Å (Figure 3b). Visualization of the Hirshfeld surfaces showed that the intermolecular interactions in the TPH crystal are mostly weak π···π-stacking and C–H···π interactions (Figure 3c). The independent gradient model based on the Hirshfeld partition (IGMH) method was applied to obtain additional insights into the noncovalent interactions between adjacent molecules (Figure 3d,e).^45,46^ In the color-coded representation of the *sign*(λ_2_)­ρ function mapped onto the isosurface in Figure 3d,e, the blue color highlights significant attractive interactions (hydrogen bonds, halogen bonds), green color corresponds to van der Waals interactions, and red color signifies notable steric repulsion or steric interactions (Figure S14). All intermolecular interactions were associated with green regions, indicating weak van der Waals interactions (*sign*(λ_2_)­ρ = [−0.02,0.02]). These weak dispersive interactions may play a role in the elastic deformation of the crystal lattice (see the discussion below).

**Figure 3:**
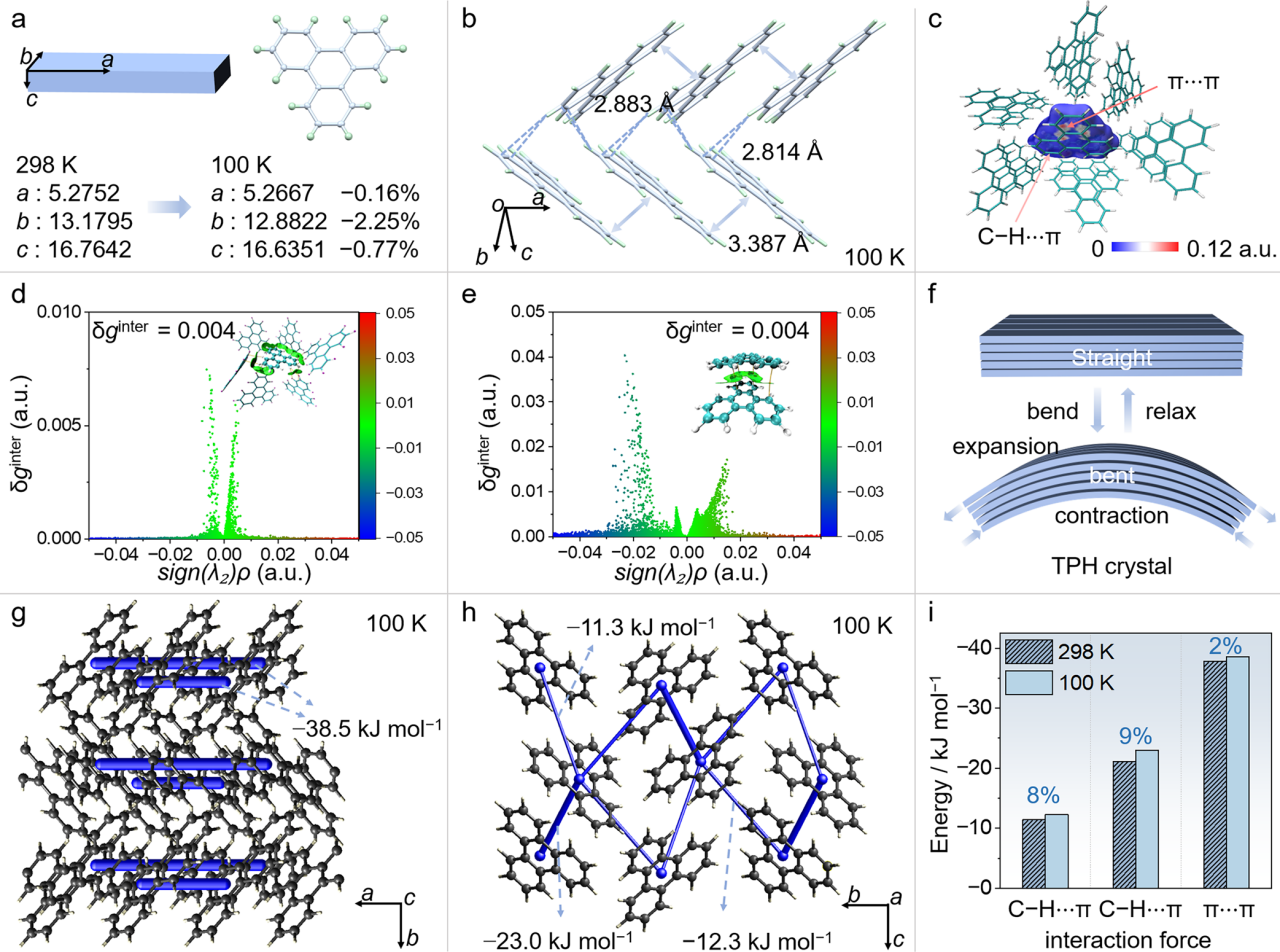
Crystal structure and intermolecular interactions. (a) Effect of temperature on the unit cell of TPH crystals between 298 and 100 K (the relative changes are given in %). (b) Prominent intermolecular interactions in the crystal at 100 K. (c) Hirshfeld surface analysis with the main intermolecular interactions highlighted. (d,e) IGMH (independent gradient model based on Hirshfeld partition) analysis of the main noncovalent interactions. (f) Diagram showing the proposed qualitative mechanism of elastic bending of a TPH crystal. The intermolecular distances are expected to increase in the outer arc and to decrease in the inner arc for a small amount due to expansion and contraction, respectively. (g,h) Plots of the energy frameworks calculated for a crystal at 100 K and shown along the *c* axis (g) and along the *a* axis (h). (i) Comparison of the energy contributions of the main interactions at 100 and 298 K. The energy change (in %) is calculated as (*E*_100 K_ – *E*_298 K_)/*E*_298 K_, where *E*_100 K_ and *E*_298 K_ are the energies at 100 and 298 K, respectively.

The face indexing confirmed that the bendable crystal face corresponds to the (011̅) plane (Figure S15). Based on the crystal structure, we hypothesize that when the crystal is subjected to a bending force in a three-point geometry, the distance between the molecules on the outer arc increases, while that between the molecules on the inner arc decreases (Figures 1b and 3f); however, confirmation of this hypothetical mechanism would require direct structure analysis that was unaffordable to us. At a rather qualitative level, we propose that intermolecular interactions can absorb and dissipate stress applied during bending, ensuring the reversibility of structural changes, and this could explain the observed elasticity (Figure S16).^47–49^ The energy frameworks of the TPH crystal at 298 and 100 K were constructed by using CrystalExplorer^50^ and the B3LYP hybrid functional with the 6-31G­(d,p) basis set^44^ where semiempirical dispersion was included by using D2 version of Grimme’s dispersion (Figures 3g,h and S17). Due to the fairly isotropic contraction of the crystal upon cooling, the weak intermolecular interactions were largely preserved at low temperatures, with only minor increases in the respective energies of −12.3 and −23.0 kJ mol^–1^ for the C–H···π interactions, and −38.5 kJ mol^–1^ for the π···π interactions at 100 K (Figures 3g–i and S17).

### Flexible Optical Waveguides of TPH Crystals

Flexible organic crystals exhibit significant potential for optical signal transmission in the visible and near-infrared regions.^51,52^ However, it has remained a significant challenge to achieve flexible organic crystals that can sustain optical waveguiding after external energy input ceases. To explore this potential of organic crystalline materials, the optical waveguiding capability of TPH crystals was studied under various conditions (Figure 4a). Leveraging the extended afterglow at 77 K, sustained phosphorescence waveguiding experiments were conducted, as illustrated in Figure 4b. The crystal was first excited with UV light, and the source was then switched off to evaluate its phosphorescent waveguiding performance. As shown in Figures 4c,d and S18a,b, the emission intensities of both straight and bent crystals at 298 and 77 K gradually decrease with increasing distance from the irradiation point. Distance-dependent emission spectra were obtained by irradiating the crystals with a 355 nm laser at various positions and collecting emission spectra from the crystal ends (Figures S18c,d and S19a,b). Using a previously reported method,^53^ the optical loss coefficients (OLCs) were calculated to be 0.296 dB mm^–1^ for straight crystal at 298 K, 0.335 dB mm^–1^ for the bent crystal at 298 K, 0.362 dB mm^–1^ for the straight crystal at 77 K, and 0.377 dB mm^–1^ for the bent crystal at 77 K (Figures S18e,f and S19c,d). As shown in Figure 4e,f, and Movie S2, the phosphorescence intensity of the TPH crystals, collected 0.5 s after turning off the UV source, gradually decreases with increasing distance from the irradiation point (Figure S20a,b). By fitting, the emissive OLC with 0.5 s duration was determined to be 0.320 dB mm^–1^ for the straight state and 0.330 dB mm^–1^ for the bent state at 77 K (Figure S20c,d). As shown in Table S8, compared to the previously reported OLCs of organic crystals, the OLC of TPH can be considered relatively high.^1,9,19,31–33,37,40,41,43,54^ The experiments confirmed that the TPH crystals exhibited remarkable abilities to transmit fluorescence and phosphorescence optical signals. Additionally, they retained their phosphorescence waveguing properties even after the external energy input was removed. This finding provides a valuable reference for designing new types of flexible “afterglow waveguides”.

**Figure 4:**
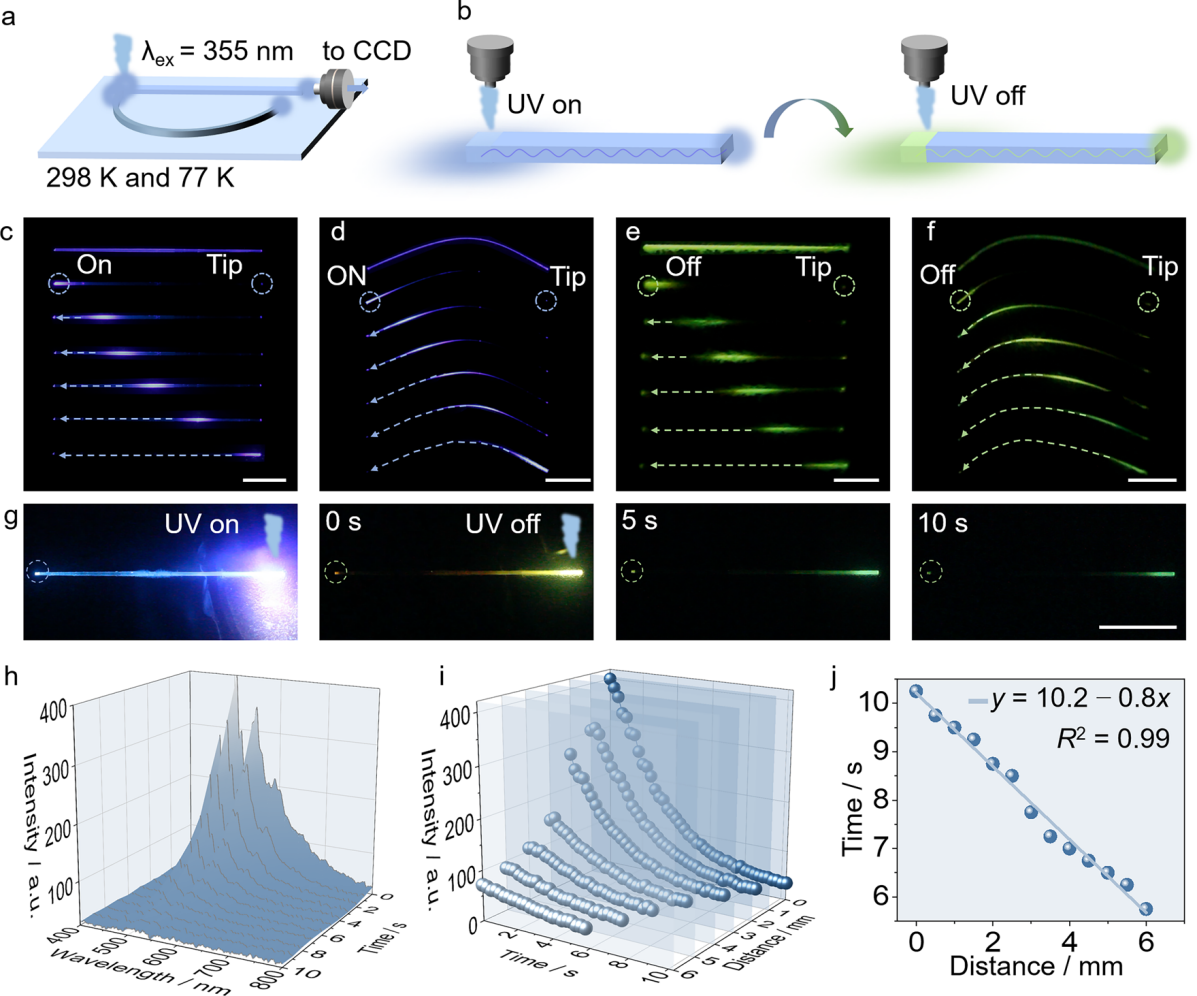
Optical signal transmission by TPH crystals. (a) Diagram of the optical waveguiding experimental setup. (b) Schematic representation of TPH crystals used as fluorescent and phosphorescent waveguides. (c,d) Photographs of a TPH crystal used as a waveguide in straight (unbent) state (c) and bent state (d) at 77 K. (e,f) Photographs of a TPH crystal used as a phosphorescent waveguide in straight (unbent) state (e) and bent state (f) at 77 K, taken 0.5 s after the UV excitation has been terminated. (g) Photographs of the TPH crystal used as a phosphorescent optical waveguide during and after UV excitation. (h) Phosphorescent emission spectra recorded over time at distance 0 mm. (i) Variation of the emission intensity, recorded at 505 nm, with spectral acquisition position and time. (j) Relationship between phosphorescence emission time and spectral acquisition position. The length of the white line scale bars in panels c–g is 2 mm.

By variation of the UV excitation position and recording of the phosphorescence signals over time, the flexible afterglow waveguide (Figure 4g) allowed for evaluation of the spatiotemporal characteristics of its phosphorescent signal transmission (Figure S21). The measurement points were selected at 0.5 mm intervals within the range of 0–6 mm. At each point, the sample was excited with 365 nm UV light, and the excitation source was then turned off to record the change of phosphorescence spectra over time. As shown in Figure 4h (Figures S22–S28), phosphorescence spectra from different excitation points were also collected and the decay curves of the phosphorescence intensity at 505 nm were plotted over time. The results demonstrated that the afterglow intensity gradually decreases over time, and both the phosphorescence emission time and intensity weaken progressively with an increasing distance from the excitation position to the collection end (Figure 4i). Linear fitting of the relationship between the distance from the excitation position to the collection end and the phosphorescence emission time revealed a linear correlation (*y* = 10.2 – 0.8*x*, Figure 4j). The results confirmed that the phosphorescence signal transmission by the TPH crystal depends on both the excitation position and the time.

### Logic Circuits and Dynamic Optical Signal Transmission of TPH Crystals

The cryogenic elasticity and long-lived phosphorescence of TPH crystals lay the foundation for the design of complex optical logic circuits, thereby unlocking the new application potential of organic crystalline materials. As shown in Figure S29, the signal intensity of the tip of most organic crystals (including TPH) decreases with increasing distance from the excitation position, exhibiting only spatial characteristics (intensity A > B).^55,56^ In contrast, the signal intensity of the phosphorescent optical waveguide made of a TPH crystal exhibits spatiotemporal characteristics; the phosphorescent output intensity of TPH can be modulated by adjusting the UV excitation position and signal acquisition time, achieving intensity relationships A > B, A = B, and A < B. Inspired by the Boolean logic operations in optical systems, we aimed at exploring the potential of programmable phosphorescent waveguides of TPH crystals for logic gate applications.

As illustrated in Figure 5a, a NOR (alternatively, NOT OR) logic gate was constructed using the phosphorescence intensity at the crystal tip as “output”, with the excitation position and signal acquisition time as two “inputs”. Specifically, phosphorescence intensity A was defined as “1” when it was greater than or equal to B, and as “0” when it was smaller than B. For instance, as shown in Figure 5b, the phosphorescence intensity A was controlled by the inputs of distance = 2 cm and time = 3 s, and the switching between 0 and 1 was achieved by varying the transduction distance and acquisition time of the phosphorescence intensity B. The phosphorescence signal intensity at 505 nm, which reflects these logic transitions, is depicted in Figure 5c. Figure S30 shows a scenario where one of the two ends of the crystal was maintained at 298 K and the other at 77 K. When UV light was shone at the end kept at 77 K, a sustained phosphorescence signal output was observed (Figure S30a,b). In contrast, when UV light was shone at the end at 298 K, no phosphorescent signal transmission was detected (Figure S30c,d), demonstrating that the long-lived transmission depends on the temperature of the excitation position (Movie S3). Building on these results, the dynamic waveguing capabilities of TPH crystals were also investigated (Figure S31). By maintaining the excitation position at 77 K and adjusting the direction of the crystal’s output signal using a needle tip, a multidirectional and completely controllable optical transmission of both fluorescence (Figure 5d and Movie S4) and phosphorescence (Figure 5e,f and Movies S5 and S6) was achieved. These experiments illustrate that the TPH crystals can be used as both fluorescence and phosphorescence optical waveguides with complete external spatiotemporal control of the light output attained by simple bending of the crystal to the desired degree.

**Figure 5:**
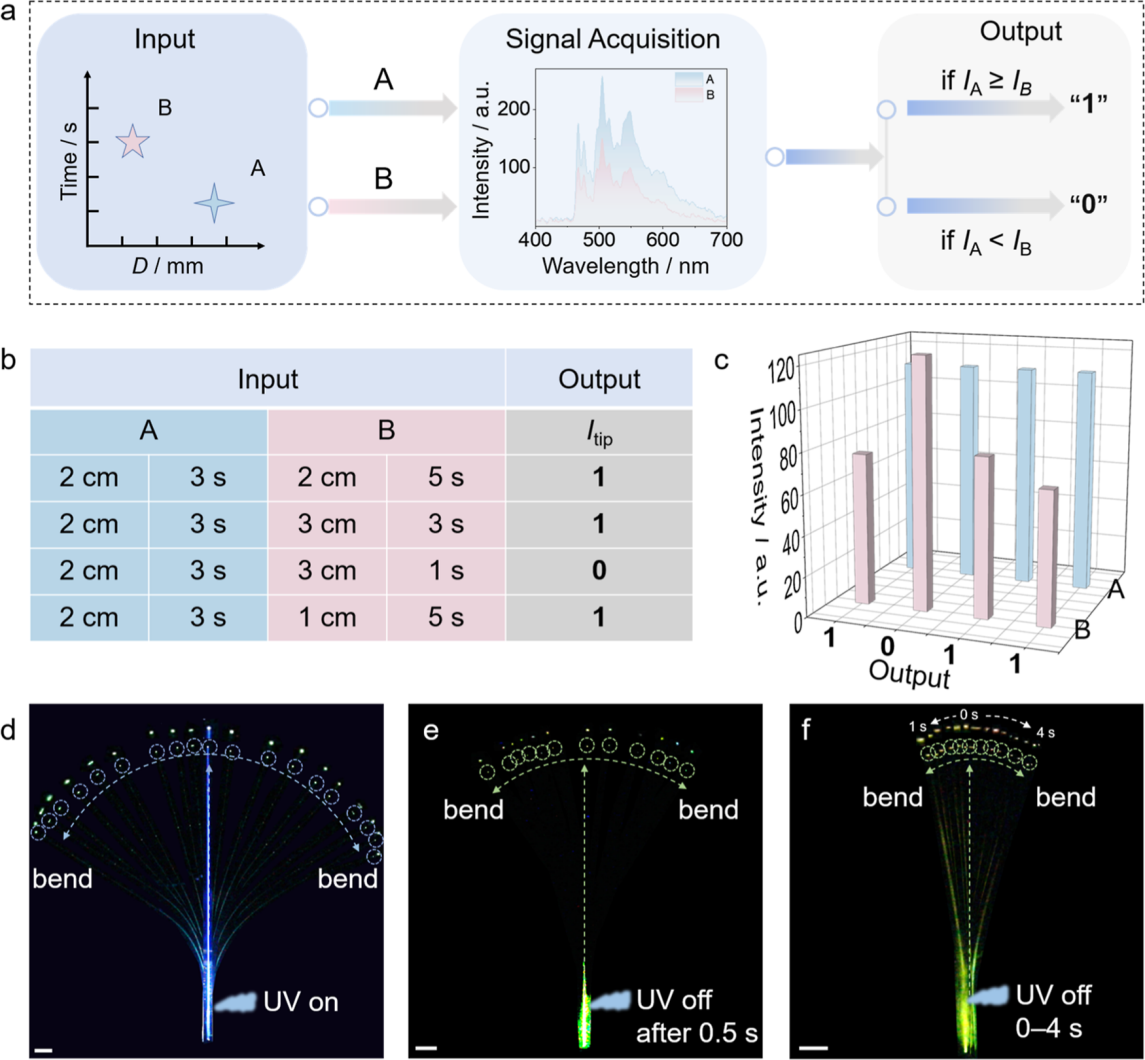
Phosphorescent dynamic optical signal transmission. (a) A transformation sequence showing the conversion of the emission output, determined by distance and time, into a binary output (*I* stands for intensity). (b) Truth table of the NOR logic gate. (c) Phosphorescence intensity emitted from the tip of the NOR logic gate at 505 nm. (d) Photographs of dynamic fluorescent optical signal transmission by the mechanically bendable TPH crystal. The light output, at a different bending angle, is highlighted with a broken circle at the top of the image. (e,f) Photographs of dynamic phosphorescent signal transmission after UV excitation of the bendable TPH crystal, under multiple 365 nm UV excitations (e) and under a single 365 nm UV excitation (f). In panel (e), each light signal output point was captured 0.5 s after the UV excitation was turned off, while panel (f) shows photos of light signal output points captured at different times immediately after the UV excitation was turned off. The length of the white scale bar in panels c–f is 2 mm. The images of the light outputs in panels d–f are enlarged for clarity and shown above the actual light outputs.

### Optical Signal Transmission by TPH Crystals through Biological Tissues

Long-lived emission from optical waveguides is central to advancing specific applications within the fields such as biosensing and optoelectronics, and organic crystals offer the unique combination of mechanical compliance, chemical versatility, and potentially biocompatibility, which are not readily accessible with inorganic optical waveguides, such as those based on silica. To explore the potential of TPH crystals for biological applications, porcine tissue samples were employed as models for transduction of light through or delivery into biological tissues (Figure 6). As shown in Figure 6a,b, when the ends of a TPH crystal were cooled with liquid nitrogen and wrapped with the tissue, a gradual decrease in the phosphorescence signal output over time (up to 10 s) was observed after UV excitation. Figures 6c illustrates the decay in the phosphorescence signal intensity over time (Movie S7). As seen in Figure 6d, the phosphorescence output was detected within the biological tissue after insertion of the TPH crystal. The light transmission was maintained even when the crystal was inserted up to 10 mm into the tissue (Figure 6e), and it illustrates the potential applications for deep-tissue irradiation.

**Figure 6:**
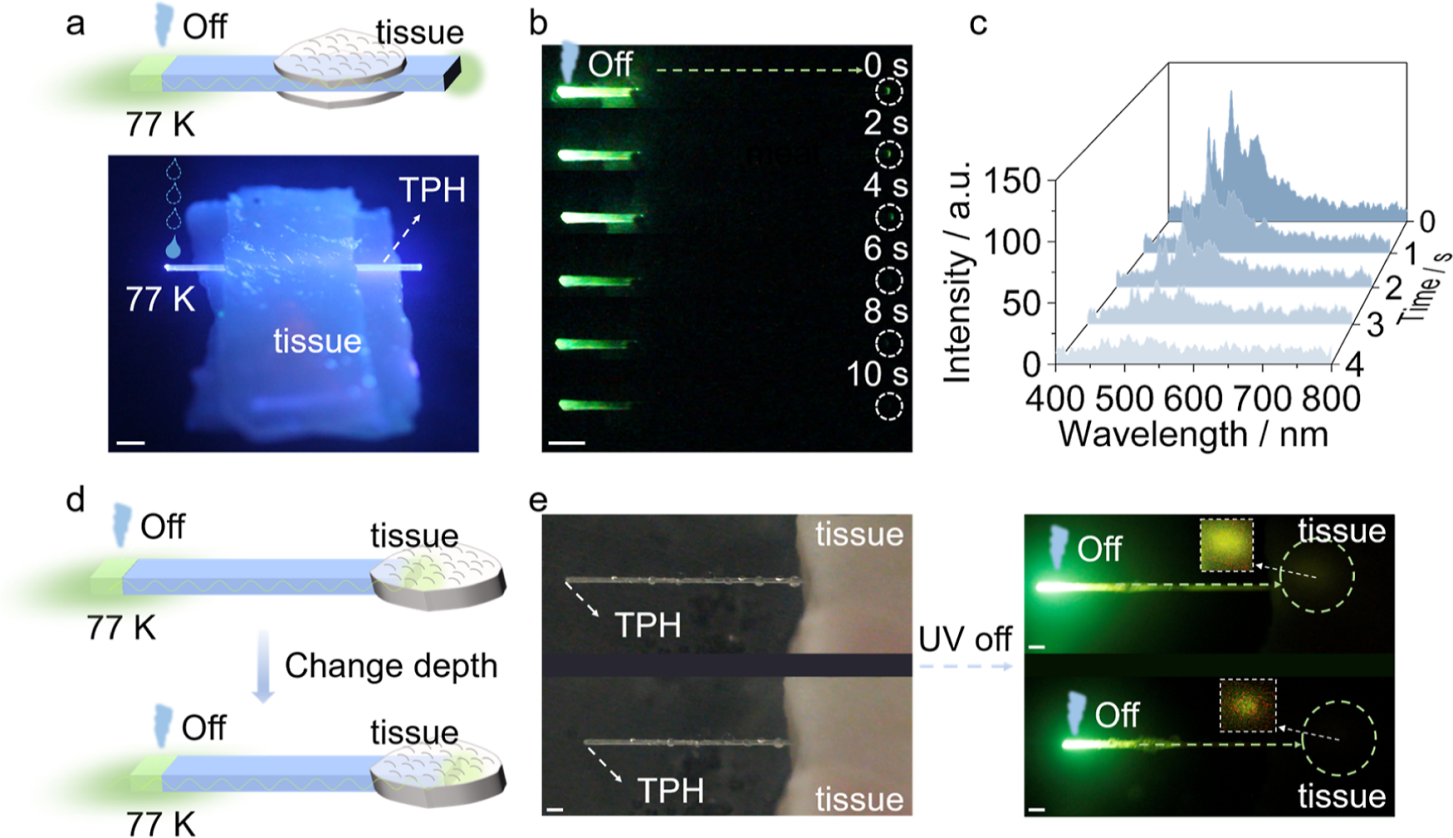
Crystals of TPH as optical signal transducers in biological tissues. (a) Schematic and photograph of a TPH crystal sandwiched between slabs of biological tissue for optical signal transmission (the crystal was cooled at its left end by applying droplets of liquid nitrogen). (b) Time-lapse photographs of the phosphorescence signal transmitted through the tissue. (c) Spectral changes in the phosphorescence emission over time recorded at the right end of the crystal in panel b. (d) Schematic showing the experiments of crystals inserted to varying depth into biological tissues for optical signal transmission. (e) Photographs of crystals inserted into tissues for transduction of phosphorescence emission. The length of the white scale bar in panels a, b and e is 2 mm.

## Conclusions

In this study, we report mechanical flexibility of an organic crystal, triphenylene (TPH), which exhibits long-lived phosphorescence lasting over 30 s, the longest duration reported for elastic organic crystals, while it retains exceptional mechanical flexibility at low temperature (liquid nitrogen). By leveraging the exceptional emissive properties of this well-known organic crystal, we achieved the integration of cryogenic elasticity and long-lived phosphorescence, enabling a synergy between mechanical and luminescent properties. The crystals of TPH were found to be exhibiting excellent fluorescence and phosphorescence waveguing at low temperatures in both straight and bent states. Moreover, the phosphorescent signal transmission displayed unique spatiotemporal characteristics, laying the foundation for a new class of optical information processing systems based on emissive organic crystal architectures. Building on this foundation, we developed phosphorescent waveguide NOR logic gates and demonstrated the optical signal transmission capabilities of TPH crystals within biological tissues. This work provides a representative example and a new direction that could overcome the challenges with combining cryogenic elasticity and long-lived phosphorescence in organic crystalline materials, a feat that was previously hindered by the opposing effects of low temperatures on mechanical and optical properties. By achieving this integration, the study addresses the critical need in the field and opens new avenues for the development of flexible low-temperature optoelectronic devices and sensors.

## Supplementary Material

















## Notes

Experimental procedures; chemical structure, ^1^H NMR spectra, and ^13^C NMR spectra of triphenylene; crystal preparation; mechanical properties of crystals; bending of the TPH crystal; characterization of the mechanical properties of crystals; three-point bending test; characterization of maximum elastic strain; cyclability testing; phosphorescence emission mechanism of crystals; photoluminescence and mechanical properties of other polycyclic aromatic compound crystals; crystal structure and molecular packing; face-indexing of a TPH crystal based on X-ray diffraction analysis; structural changes in crystal bending; energy framework calculations; optical waveguiding characterization; phosphorescent optical signal transmission (PDF)
Change in the phosphorescence emission from a TPH crystal while it is being bent (MP4)
Waveguiding of the delayed phosphorescence through a straight TPH crystal (MP4)
Temperature dependence of the waveguiding capability (MP4)
Spatial control over the emission output (MP4)
Spatial control over multiple light output (MP4)
Spatial control over the light output (MP4)
Phosphorescence waveguiding of TPH crystals in a biological tissue (MP4)
