# Supplementary material for: Cryogenically Flexible Phosphorescent Organic Crystals that Transmit Self-Sustained Persistent Luminescence with Spatiotemporal Control
Source: J Am Chem Soc. 2025 Jun 16;147(26):22961–71. doi: 10.1021/jacs.5c05733 (PMC12232176; doi:10.1021/jacs.5c05733)
Supplement: Supplementary file 1 [file ja5c05733_si_001.pdf]

Supporting Information

Cryogenically Flexible Phosphorescent Organic Crystals that Transmit Self-Sustained Persistent Luminescence with Spatiotemporal Control

Xuesong Yang,[1] Mingqi Zhang,[1] Baolei Tang,[1] Lijie Wang,[2] Bing Yang,[1] Liang Li,[3,4] Panče Naumov,[4,5,6,7*] and Hongyu Zhang[1*]

[1]*State Key Laboratory of Supramolecular Structure and Materials, College of Chemistry, Jilin University, Changchun 130012, P. R. China*

[2]*State Key Laboratory of Integrated Optoelectronics, College of Electronic Science and Engineering, Jilin University, Changchun, 130012, China*

[3]*SAFIR Novel Materials Development Lab, Sorbonne University Abu Dhabi, PO Box 38044, Abu Dhabi, UAE*

[4]*Smart Materials Lab, New York University Abu Dhabi, PO Box 129188, Abu Dhabi, UAE*

[5]*Center for Smart Engineering Materials, New York University Abu Dhabi, PO Box 129188, Abu Dhabi, UAE*

[6]*Research Center for Environment and Materials, Macedonian Academy of Sciences and Arts, Bul. Krste Misirkov 2, MK–1000 Skopje, Macedonia*

[7]*Molecular Design Institute, Department of Chemistry, New York University, 100 Washington Square East, 10003, New York, USA*

**Corresponding authors.*

Supplementary Methods

General information. All solvents and starting materials for synthesis were purchased from commercial sources. ^{1}H and $^{13}C\{^{1}H\}$ NMR spectra were recorded on a Bruker Avance 400 MHz spectrometer with tetramethylsilane as internal standard. Elemental analyses were performed on an Elementar Vario Micro Cube analyzer. The crystals were observed by using an Olympus BX61 microscope. Three-point bending and tensile tests were carried out using an Instron 5944 universal testing system with a capacity of 5 N Instron 2530 load cell. For the optical waveguiding tests, the crystals were irradiated by the third harmonic (355 nm) of a Nd:YAG (yttrium-aluminum-garnet) laser at a repetition rate of 10 Hz and pulse duration of about 10 ns. The energy of the laser was adjusted by using the calibrated neutral density filters. The beam was focused on a strip whose shape was adjusted to 3.3 × 0.6 mm by using a cylindrical lens and a slit. The emission spectra were recorded on an Ocean Insight Maya2000 Pro CCD spectrometer. Scanning electron microscopy (SEM) images were obtained on the FEI Quanta 450 operated at 5–10 kV. Phosphorescence lifetimes were determined by Edinburgh Instruments FLS980-S2S2-stm spectrometer. The fluorescence and phosphorescence quantum yields were determined using the OXFORD Microstat/FLS980 spectrometer.

X-ray crystallographic analysis. Diffraction data were collected on a Rigaku R-AXIS RAPID diffractometer. The data collection, integration, scaling, and absorption corrections were performed by using the Bruker Apex 3 software.[1] The structures were solved with direct methods using Olex2,[2] and refined by using the full-matrix least-squares method on F^2. The non-hydrogen atoms were refined anisotropically. The positions of the hydrogen atoms were calculated and refined isotropically. The program PLATON was used for the geometric calculations.[3] The graphics related to the structures were generated by using Mercury 4.2.0.[4] The crystallographic

information has been deposited at the Cambridge Crystallographic Data Centre (CCDC) and can be retrieved with the CCDC numbers 2389555 (100 K) and 2389556 (298 K).

Characterization. Triphenylene was purchased from Energy Chemical. ^{1}H NMR (chloroform-*d*, 400 MHz) δ 8.71 – 8.55 (m, 6H), 7.65 (dq, J = 6.6, 3.1 Hz, 6H). ^{13}C NMR (chloroform-*d*, 101 MHz) δ 129.84, 127.28, 123.36. MS (ESI+): m/z calcd for $C_{18}H_{12}$ $[M+H]^{+}$: 228.09; Found: 228.15. Anal. calcd (%) for $C_{18}H_{12}$: C, 94.70; H, 5.30. Found: C, 94.78; H, 5.28.

Supplementary Figures

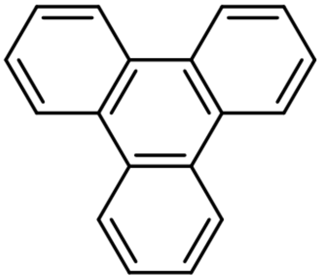

Figure S1. Chemical structure of triphenylene (TPH).

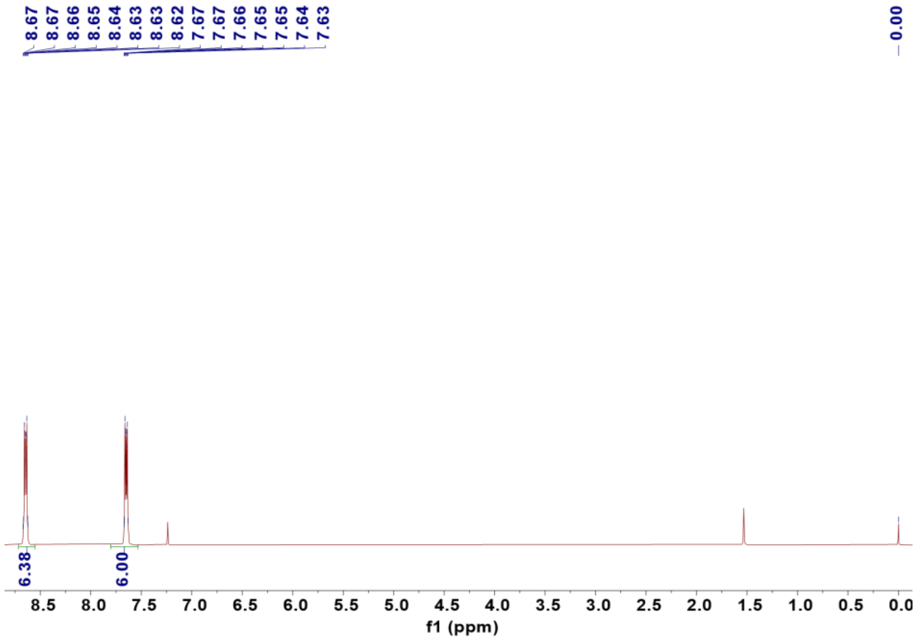

Figure S2. ^{1}H NMR spectrum of TPH (400 MHz, chloroform-*d*).

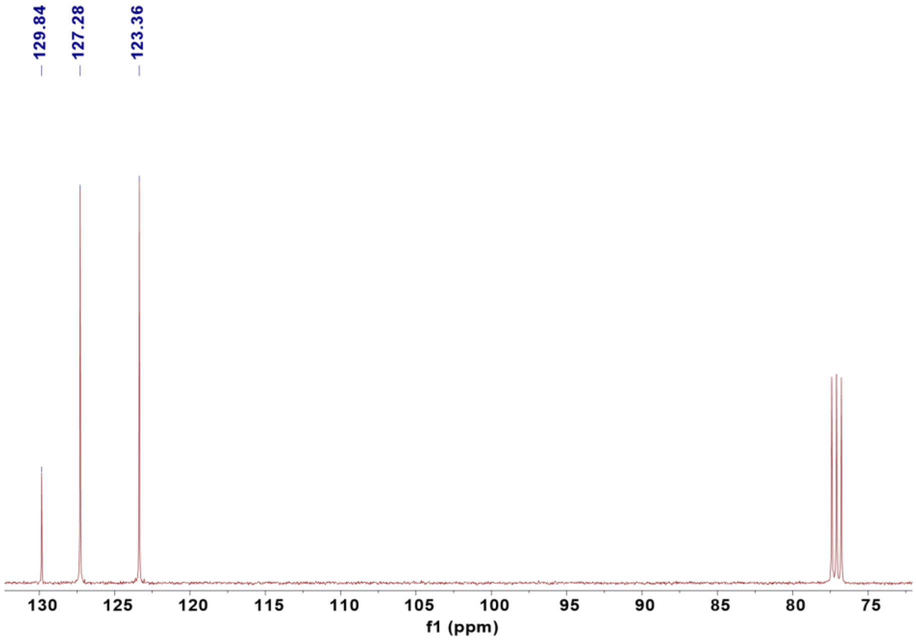

Figure S3. ^{13}C NMR spectrum of TPH (101 MHz, chloroform-*d*).

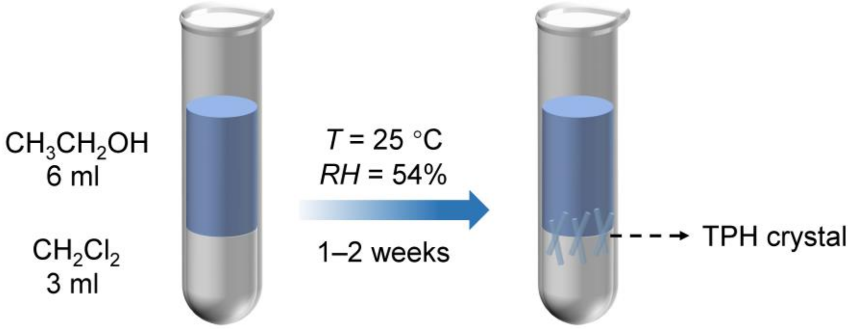

Figure S4. Crystal preparation. Schematic diagram showing the method used for growing TPH crystals. The dichloromethane solution was saturated.

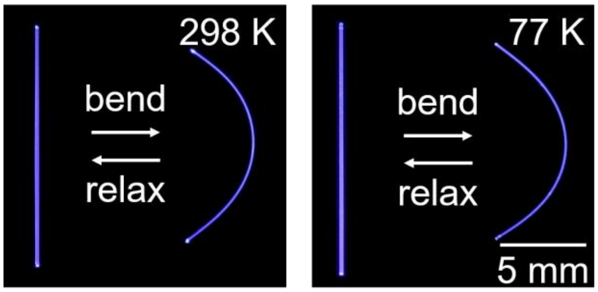

Figure S5. Mechanical properties of crystals. Photographs of reversible bending of TPH crystals at 298 K and 77 K.

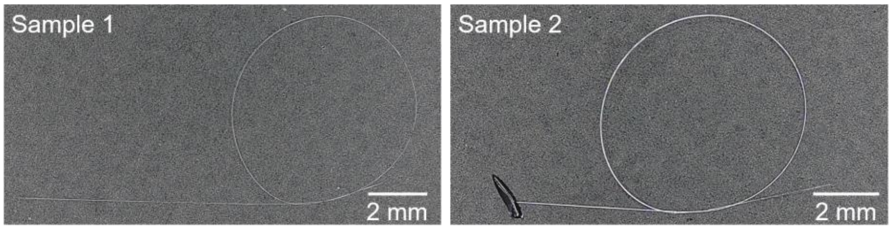

Figure S6. Bending of the TPH crystal. Photographs of the TPH crystal wound into a ring.

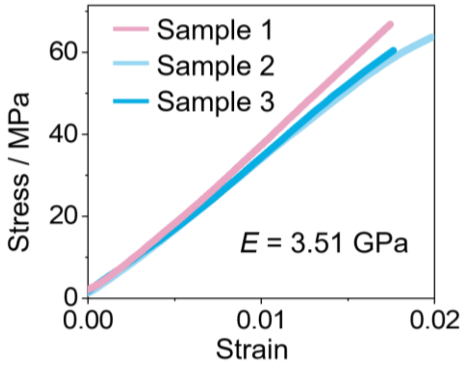

Figure S7. Characterization of the mechanical properties of crystals. Stress-strain profiles of TPH obtained by the three-point bending test.

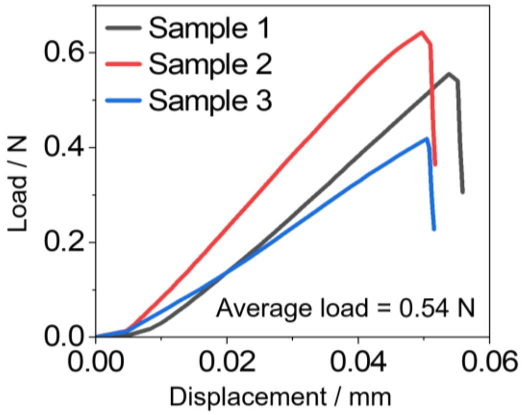

Figure S8. Three-point bending test. The displacement-load curves of TPH crystals for different samples, with the curve's abrupt change indicating the crystal fracture location. Sample 1 (thickness: 0.255 mm, width: 0.382 mm), Sample 2 (thickness: 0.293 mm, width: 0.353 mm), and Sample 3 (thickness: 0.257 mm, width: 0.314 mm).

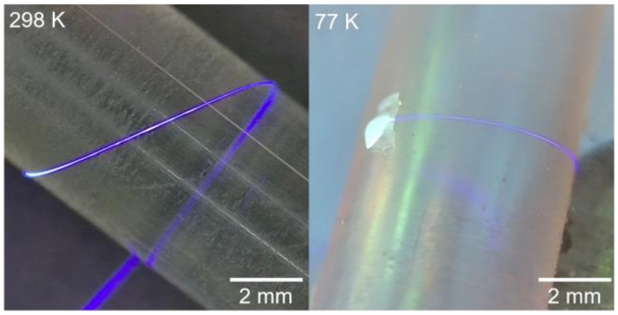

Figure S9. Characterization experiments of maximum elastic strain. Photographs of the maximum elastic strain of the crystals tested at 298 K and 77 K.

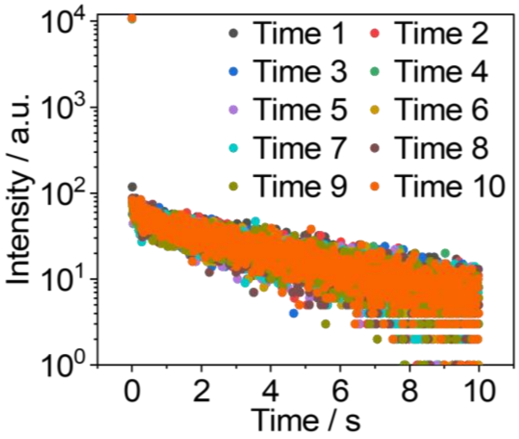

Figure S10. Cyclability tests. Intensity decay curves are shown for TPH crystals at 77 K excited multiple times.

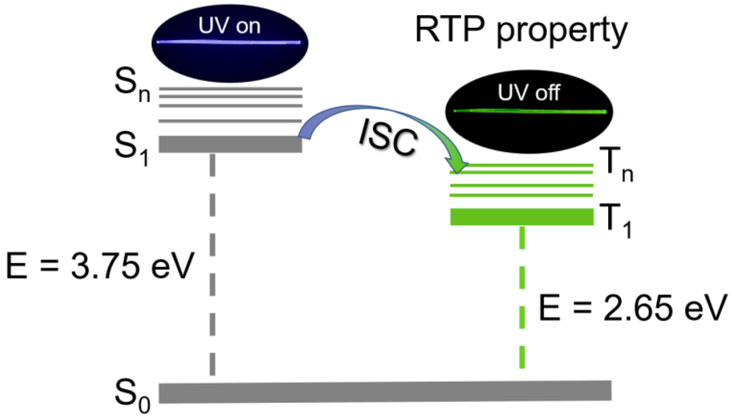

Figure S11. Mechanism of the phosphorescence emission of the crystals. A Jablonski diagram showing the suggested phosphorescence emission mechanism of TPH crystals.

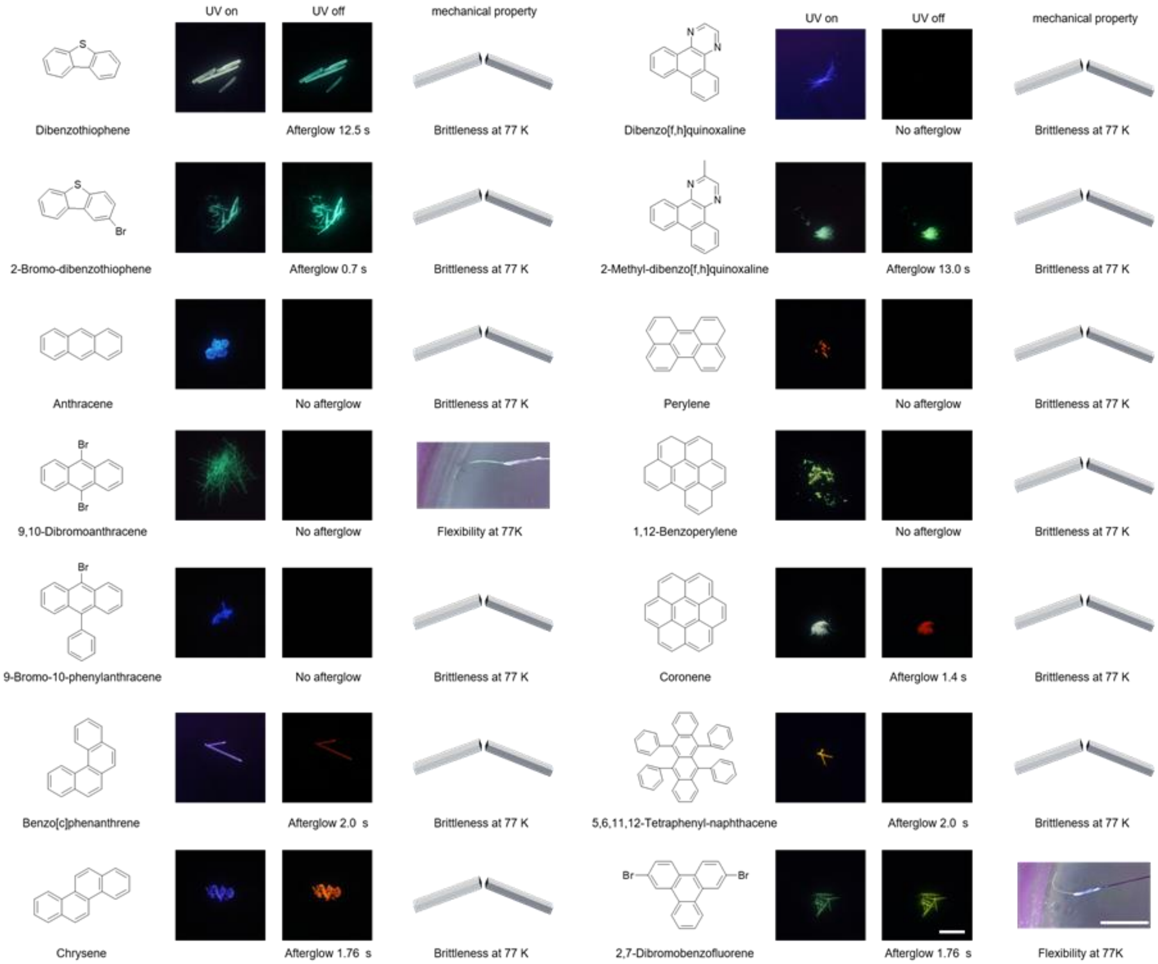

Figure S12. Photoluminescence and mechanical properties of polycyclic aromatic compound crystals. Mechanical properties and phosphorescence characteristics of polycyclic aromatic compounds with different chemical structures at low temperature (77 K). The length of the white scale bar is 5 mm.

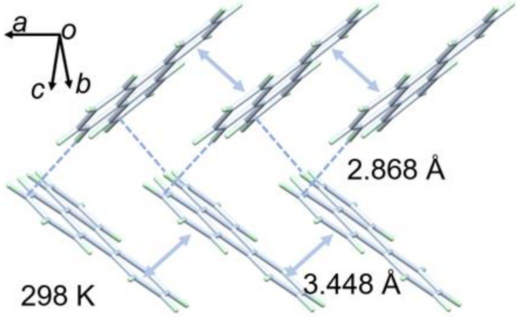

Figure S13. Crystal structure and molecular packing. Molecular stacking diagram of the TPH crystal along the crystallographic [100] direction at 298 K.

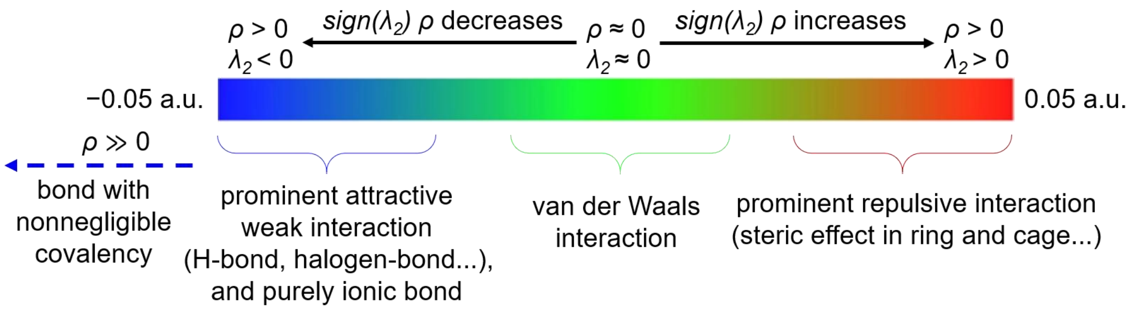

Figure S14. Common interpretation of the coloring method of mapped function *sign*(λ_2)ρ in IGMH maps.

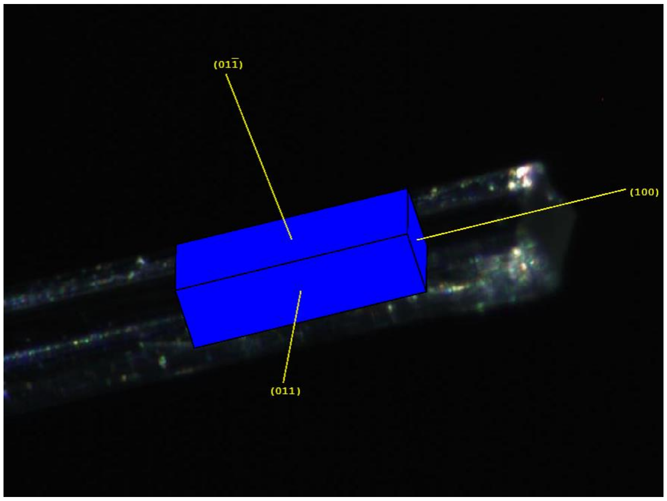

Figure S15. Face-indexing of a TPH crystal based on X-ray diffraction.

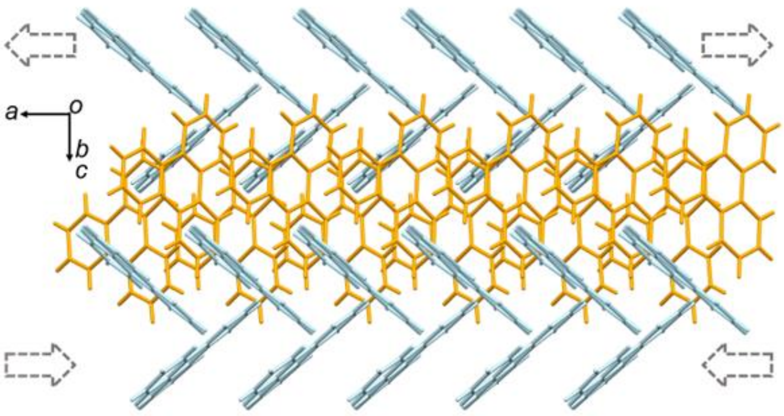

Figure S16. Structural changes in crystal bending. Parallel stacking structure, showing expansion and contraction of the inner and outer crystal arcs during bending.

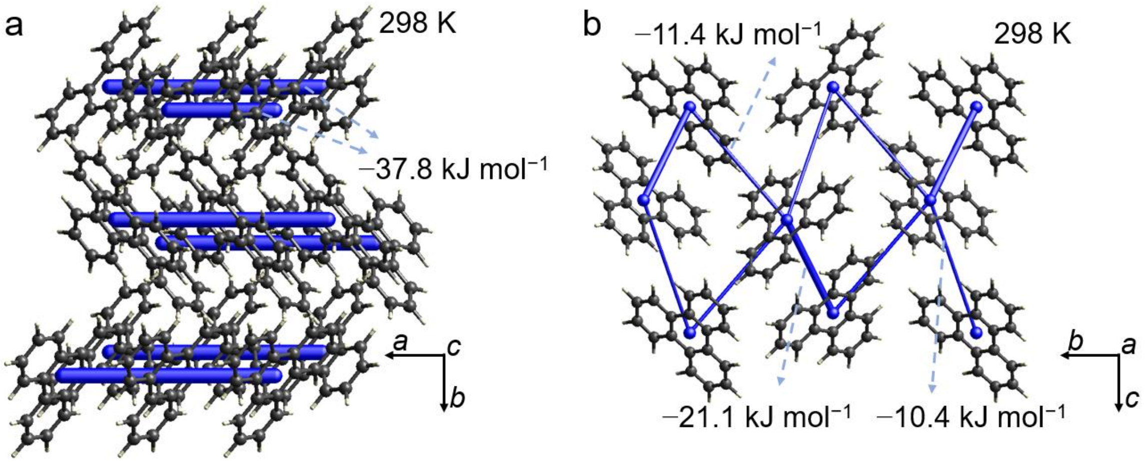

Figure S17. Energy framework calculations. Energy frameworks for TPH are shown along the *c*-axis (a) and the *a*-axis (b) at 298 K.

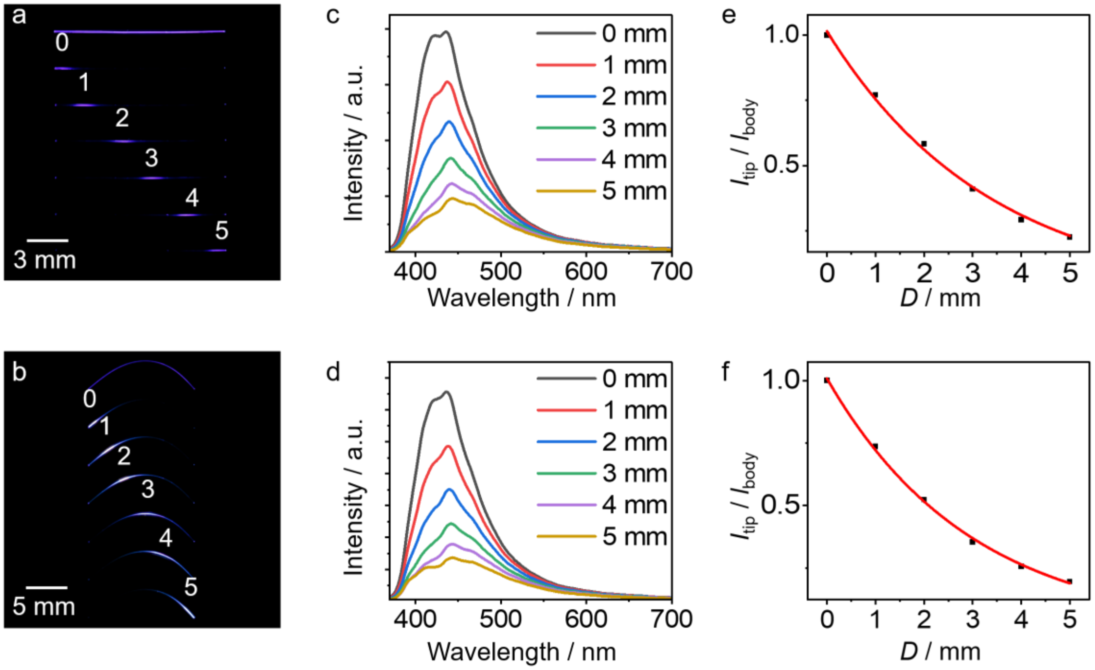

Figure S18. Optical waveguiding. (a,b) Image of TPH used as a waveguide in straight state (a) and bent state (b) at 298 K. (c,d) Fluorescence spectra were collected at the fixed end of the crystal, while the crystals were exited at different position by 355 nm laser, position differences between the fixed end and the excitation position was defined as distance (mm) at 298 K. The panels c and d correspond to the crystals shown in panels a and b respectively. (e,f) Decay of intensity with distance I_{tip}/I_{body} at 298 K. The optical loss coefficient (α) was obtained by a single exponential fitting function. $I_{tip}/I_{body} = A\exp(-\alpha D)$, in which I_{tip} and I_{body} are the fluorescence intensities measured at the fixed end and the excitation position, respectively. A is the optical loss coefficient and D is position differences between the fixed end and the excitation position. The panels showed in straight state (e) and bent state (f).

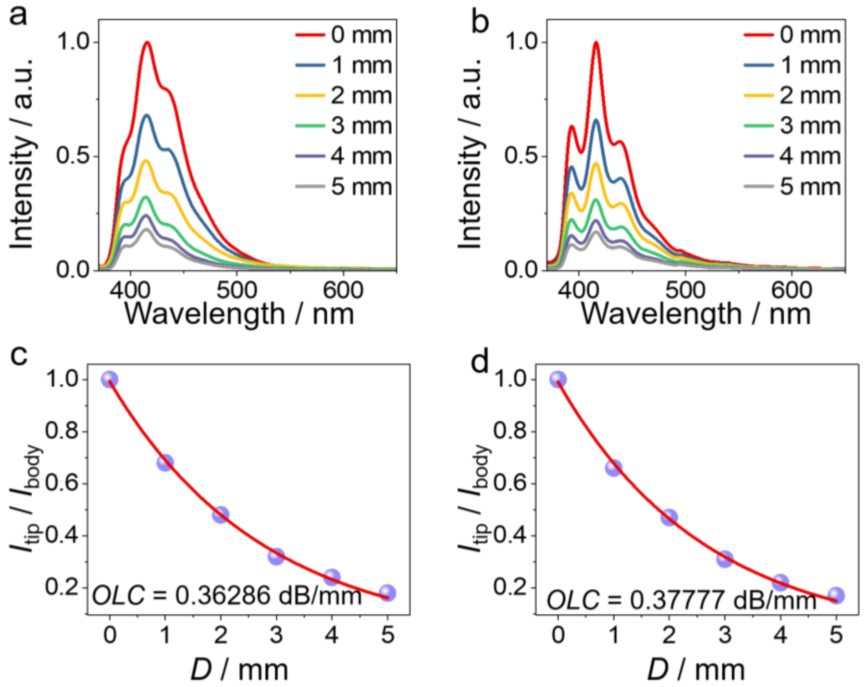

Figure S19. Optical waveguiding characterization. (a,b) Fluorescence spectra were collected at the fixed end of the crystal, while the crystals were excited at different position by a 355 nm laser. The position differences between the fixed end and the excitation position is defined as the distance (mm) at 77 K. The panels a and b were corresponding to the crystals shown in panels Figure 4c and d respectively. (c,d) Fluorescence intensity decay with distance I_{tip}/I_{body} for TPH crystals in the straight (c) and bent (d) states at 77 K, corresponding to (a) and (b), respectively.

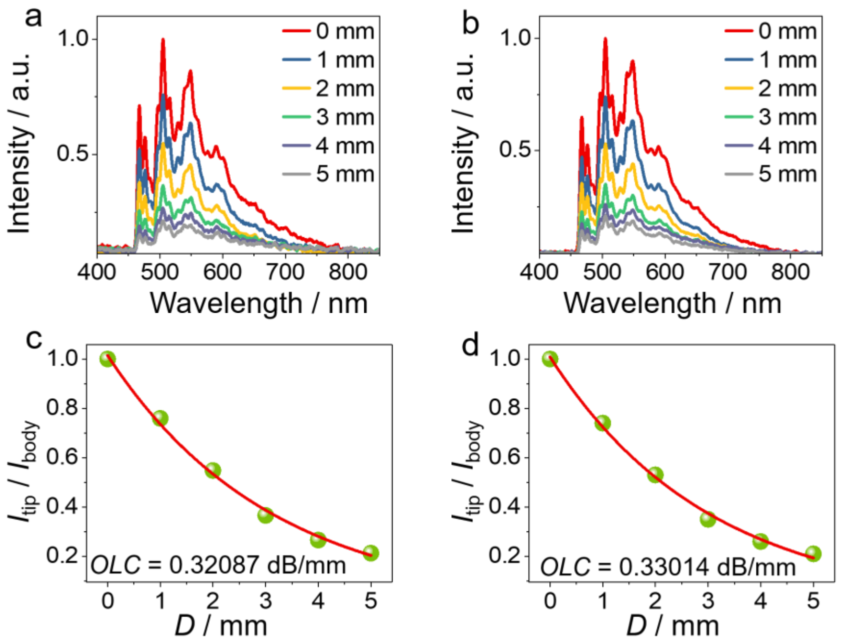

Figure S20. Optical waveguiding characterization. (a,b) Phosphorescence spectra were collected at the fixed end of the crystal, while the crystals were excited at different position by 355 nm laser, position differences between the fixed end and the excitation position is defined as distance (mm) at 77 K. The panels a and b correspond to the crystals shown in panels Figure 4e and f respectively. (c,d) Phosphorescence intensity decay with distance I_{tip}/I_{body} for TPH crystals in the straight (c) and bent (d) states at 77 K, corresponding to (a) and (b), respectively.

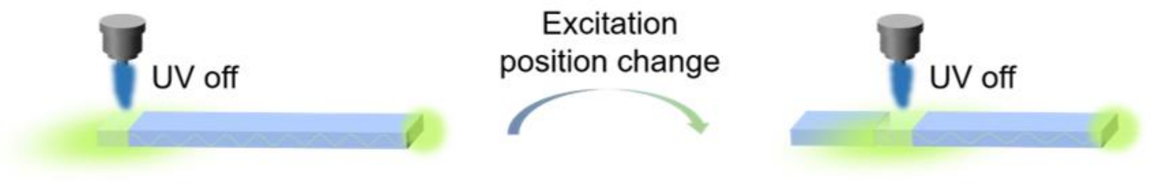

Figure S21. Spatiotemporal characteristics of the phosphorescent signal transmission. Schematic of the spatiotemporal properties testing for TPH crystals phosphorescent waveguides.

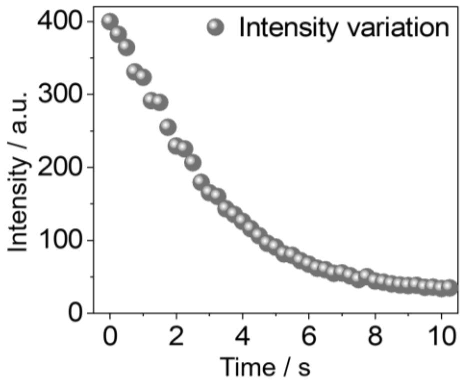

Figure S22. Phosphorescent optical signal transmission. Plot of 505 nm emission wavelength intensity versus time at 0 mm.

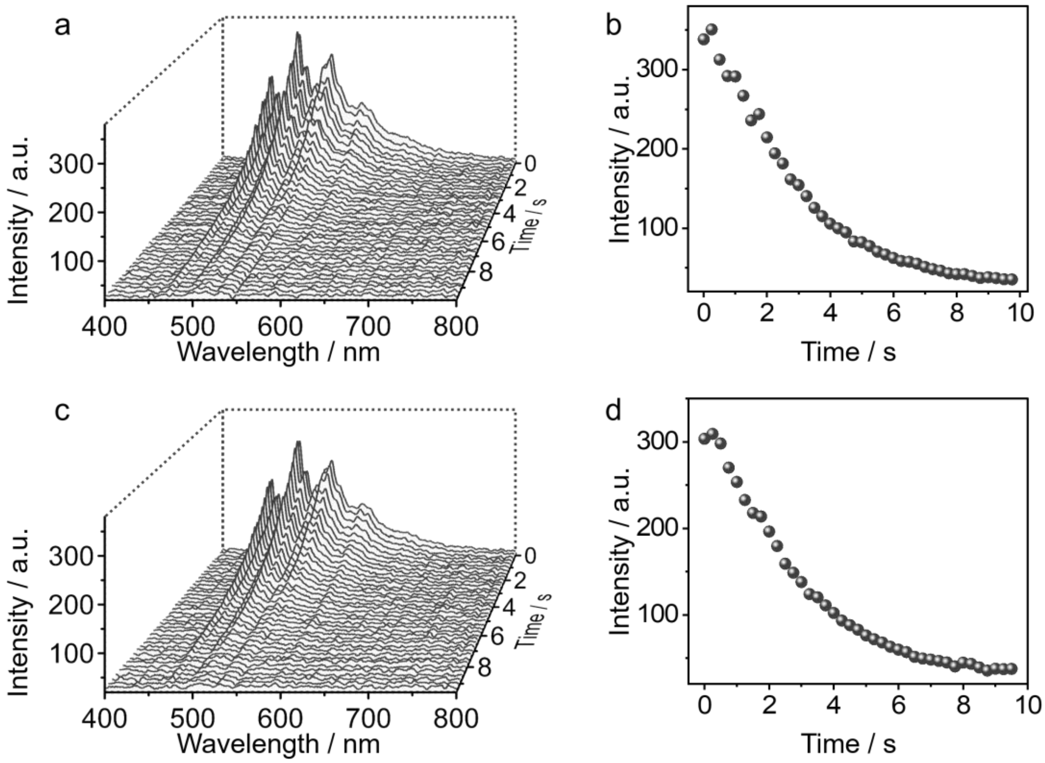

Figure S23. Phosphorescent optical signal transmission. (a) Phosphorescent emission spectra over time collected at 0.5 mm. (b) Plot of 505 nm emission wavelength intensity versus time at 0.5 mm. (c) Phosphorescent emission spectra over time collected at 1.0 mm. (d) Plot of 505 nm emission wavelength intensity versus time at 1.0 mm.

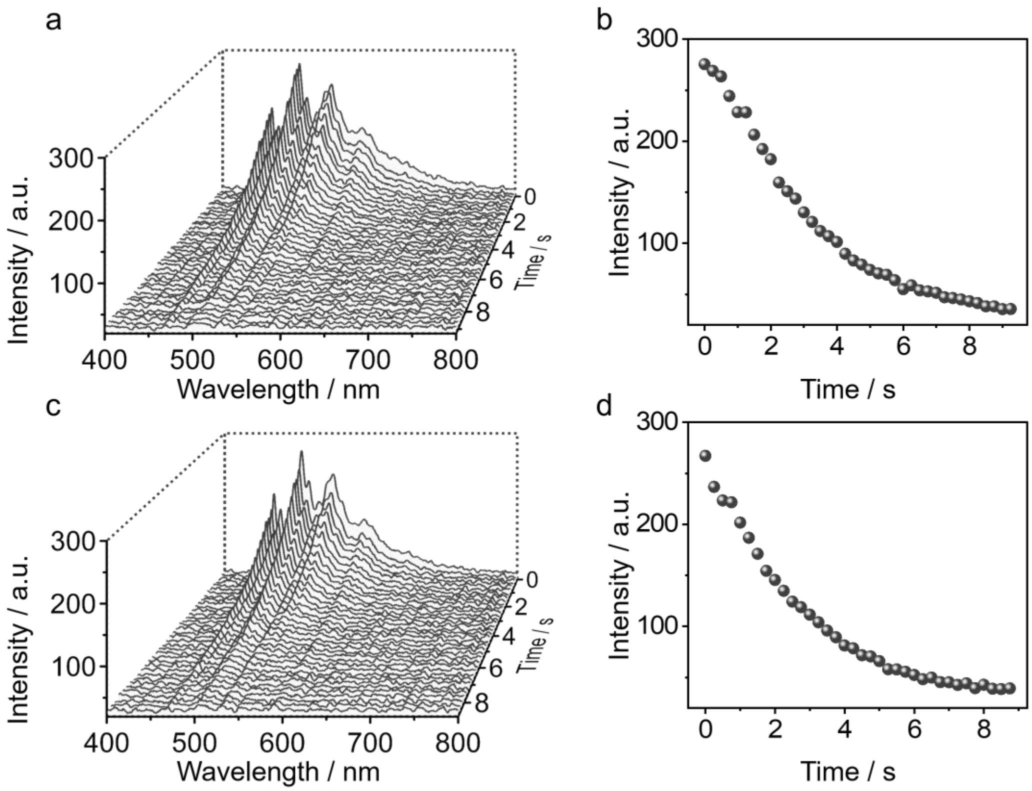

Figure S24. Phosphorescent optical signal transmission. (a) Phosphorescent emission spectra over time collected at 1.5 mm. (b) Plot of 505 nm emission wavelength intensity versus time at 1.5 mm. (c) Phosphorescent emission spectra over time collected at 2.0 mm. (d) Plot of 505 nm emission wavelength intensity versus time at 2.0 mm.

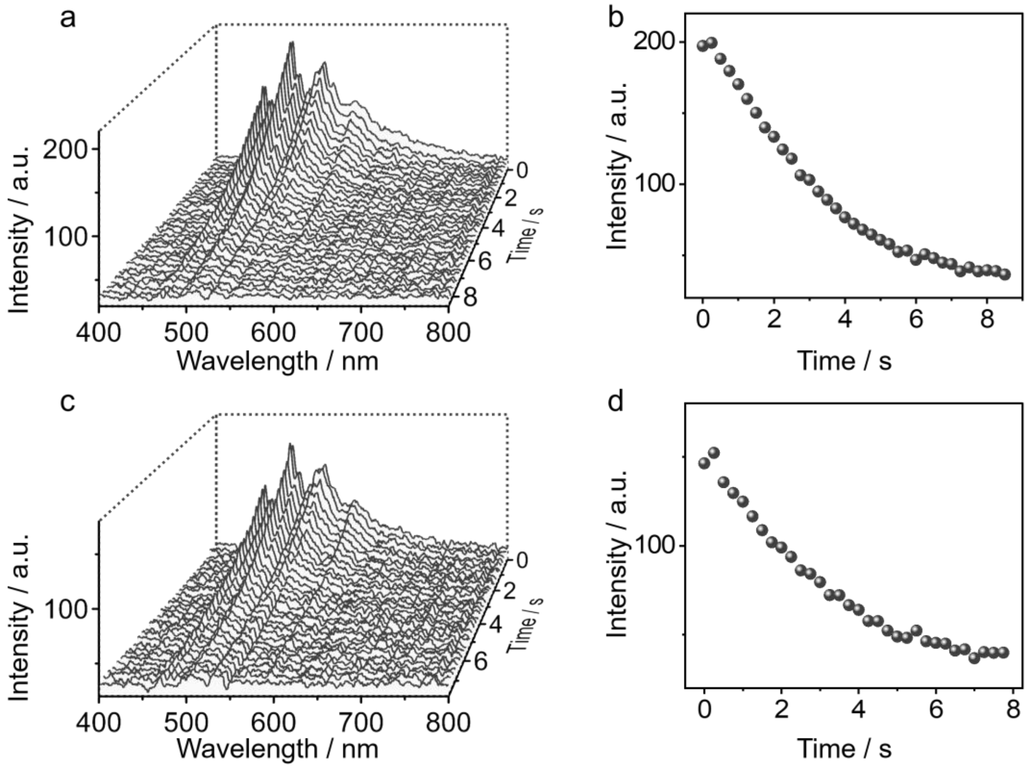

Figure S25. Phosphorescent optical signal transmission. (a) Phosphorescent emission spectra over time collected at 2.5 mm. (b) Plot of 505 nm emission wavelength intensity versus time at 2.5 mm. (c) Phosphorescent emission spectra over time collected at 3.0 mm. (d) Plot of 505 nm emission wavelength intensity versus time at 3.0 mm.

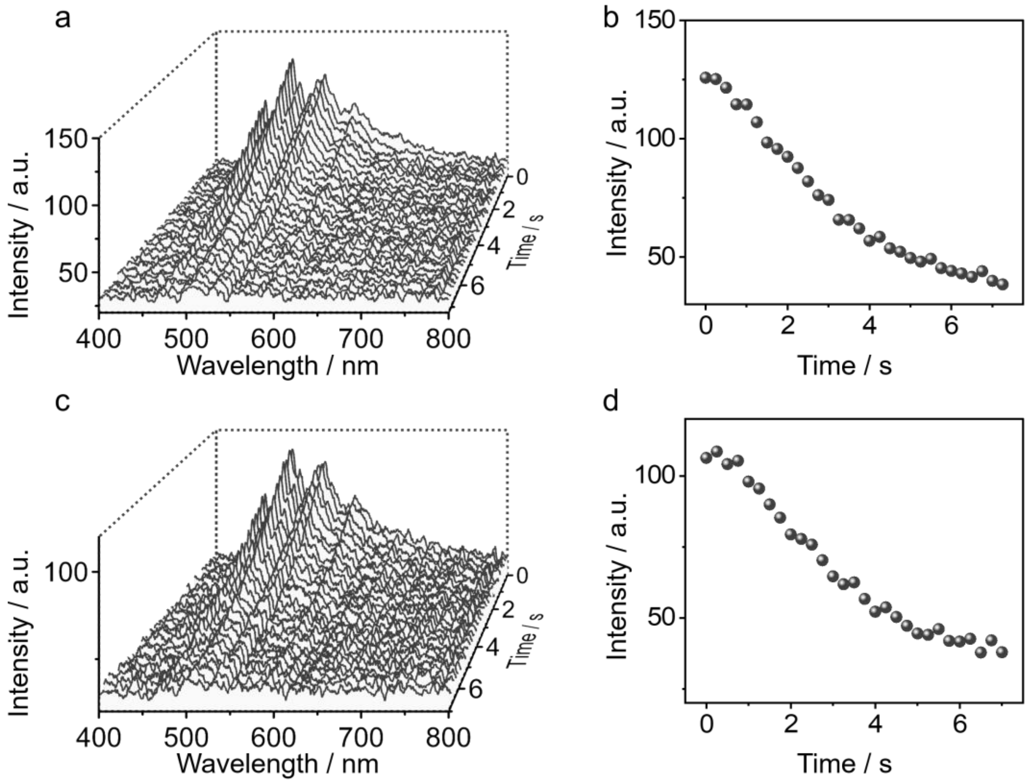

Figure S26. Phosphorescent optical signal transmission. (a) Phosphorescent emission spectra over time collected at 3.5 mm. (b) Plot of 505 nm emission wavelength intensity versus time at 3.5 mm. (c) Phosphorescent emission spectra over time collected at 4.0 mm. (d) Plot of 505 nm emission wavelength intensity versus time at 4.0 mm.

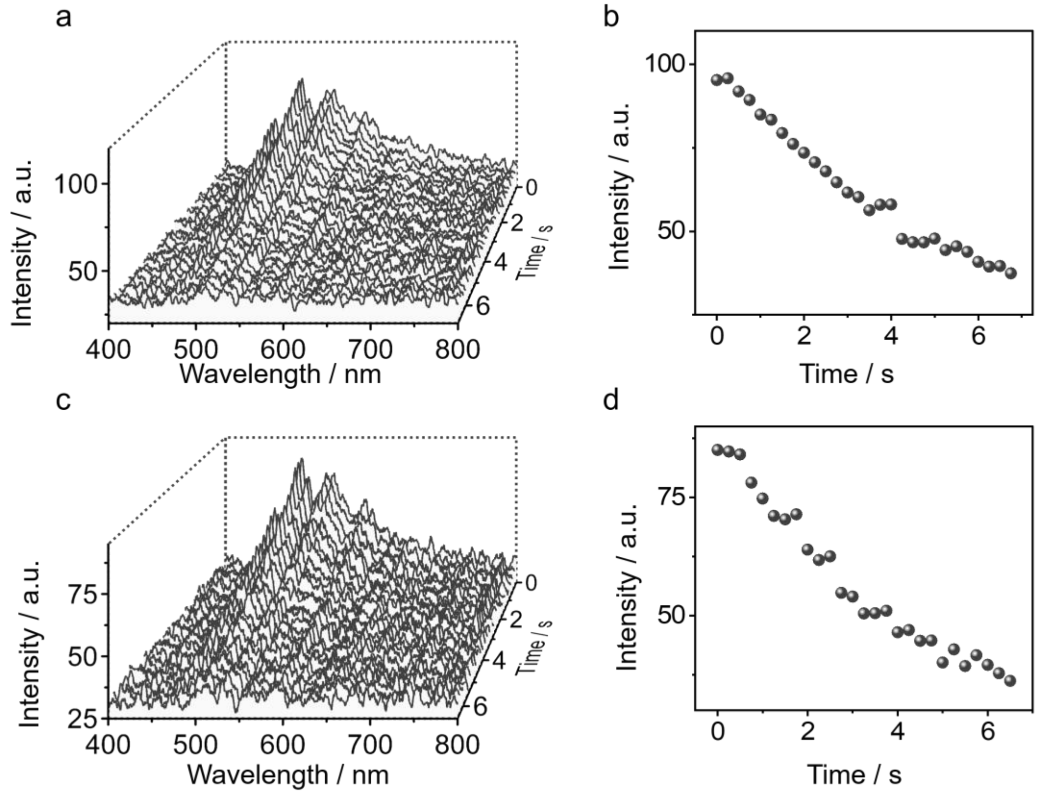

Figure S27. Phosphorescent optical signal transmission. (a) Phosphorescent emission spectra over time collected at 4.5 mm. (b) Plot of 505 nm emission wavelength intensity versus time at 4.5 mm. (c) Phosphorescent emission spectra over time collected at 5.0 mm. (d) Plot of 505 nm emission wavelength intensity versus time at 5.0 mm.

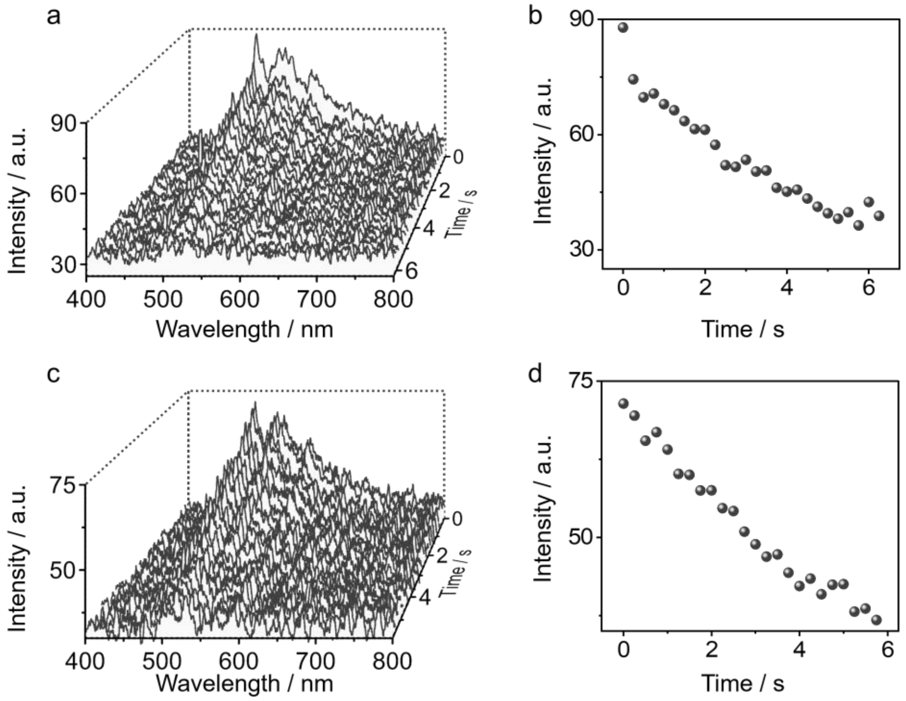

Figure S28. Phosphorescent optical signal transmission. (a) Phosphorescent emission spectra over time collected at 5.5 mm. (b) Plot of 505 nm emission wavelength intensity versus time at 5.5 mm. (c) Phosphorescent emission spectra over time collected at 6.0 mm. (d) Plot of 505 nm emission wavelength intensity versus time at 6.0 mm.

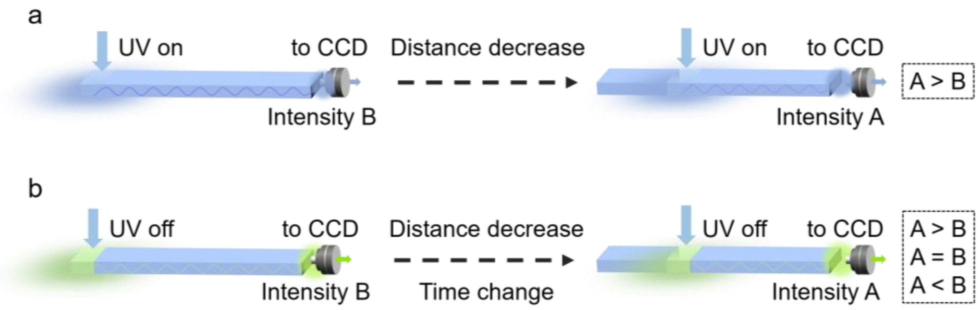

Figure S29. Optical signal transmission. (a) Schematic showing the effect of distance on TPH crystals fluorescent waveguide signal intensity. (b) Schematic illustrating the effects of distance and time on the phosphorescent signal intensity in the TPH crystal waveguide.

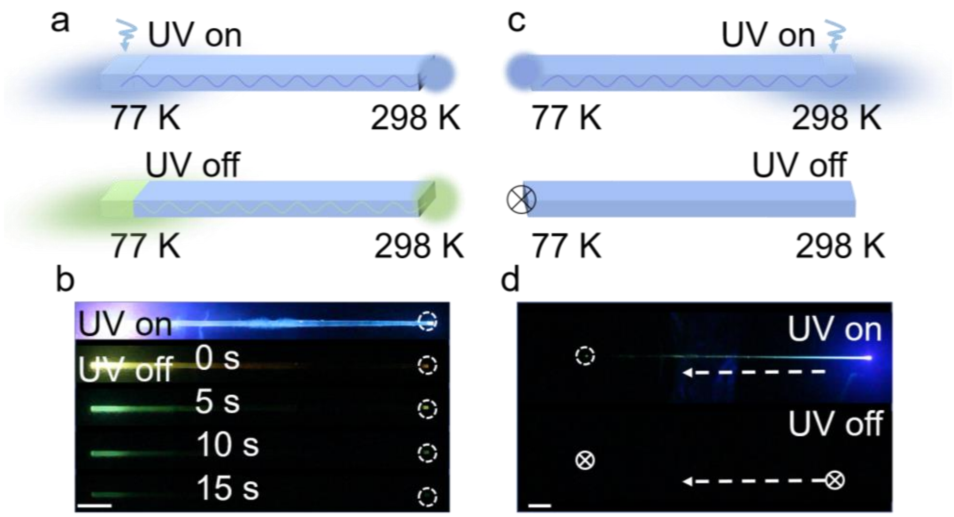

Figure S30. Phosphorescent optical signal transmission. (a) Schematic of phosphorescent optical signal transmission when one end of the crystal was at 298 K and the other at 77 K, with excitation of the 77 K end. (b) Photographs of phosphorescent optical signal transmission corresponding to (a). (c) Schematic of phosphorescent optical signal transmission when one end of the crystal was at 298 K and the other at 77 K, with excitation at the 298 K end. (d) Photographs of optical signal transmission corresponding to (c). The length of the white line scale bar is 2 mm.

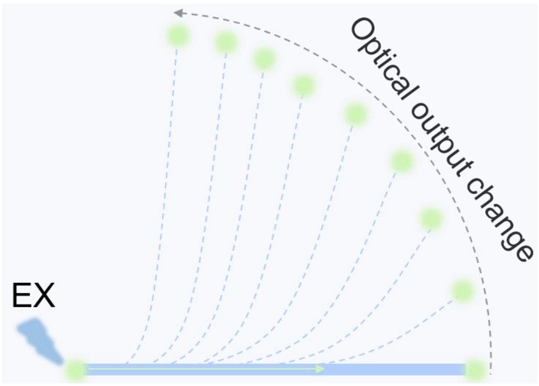

Figure S31. Dynamic optical signal transmission. Schematic diagram of crystal multi-directional optical signal output.

Supplementary Tables

Table S1. Relationship between crystal thickness and the maximum elastic strain ε / %= (t / (t + d)) * 100% (the crystals that cracked are highlighted in red color)

t / mm (298 K)	d / mm (298 K)	ε / % (298 K)	t / mm (77 K)	d / mm (77 K)	ε / % (77 K)
0.017	6.17	0.27	0.016	6.17	0.26
0.026	6.17	0.42	0.018	6.17	0.29
0.031	6.17	0.50	0.021	6.17	0.34
0.04	6.17	0.64	0.025	6.17	0.40
0.051	6.17	0.82	0.027	6.17	0.44
0.056	6.17	0.90	0.031	6.17	0.50
0.062	6.17	0.99	0.035	6.17	0.56
0.068	6.17	1.09	0.038	6.17	0.61
0.072	6.17	1.15	0.041	6.17	0.66
0.076	6.17	1.22	0.044	6.17	0.71
0.078	6.17	1.25	0.047	6.17	0.76
0.08	6.17	1.28	0.049	6.17	0.79
0.082	6.17	1.31	0.053	6.17	0.85
0.085	6.17	1.36	0.055	6.17	0.88
0.088	6.17	1.41	0.058	6.17	0.93
0.09	6.17	1.44	0.061	6.17	0.98
0.091	6.17	1.45	0.064	6.17	1.03

0.092	6.17	1.47	0.066	6.17	1.06
0.093	6.17	1.48	0.067	6.17	1.07
0.094	6.17	1.50	0.068	6.17	1.09
0.095	6.17	1.52	0.069	6.17	1.11
ε / %	1.48		ε / %	1.07	

Table S2. Comparison of elastic modulus of TPH crystals with previously reported flexible organic crystals

Name	Young's modulus	Ref
Cry-1R	0.65 GPa	37
S-BPEMP	0.74 GPa	33
DPVB	0.85 GPa	39
compound 1	1.60 GPa	31
DPPC	2.30 GPa	40
P-2	2.45 GPa	41
HDBP	3.50 GPa	9
P-1	3.51 GPa	41
**TPH**	**3.51 GPa**	**This work**
HMBPPA	4.00 GPa	29
CRY-G	6.60 GPa	42
m-DPTPD	6.88 GPa	43
CRY-Y	8.10 GPa	42
p-DPTPD	10.45 GPa	43
HDED	12.00 GPa	32

Table S3. Calculated vertical excitation energies, absorption wavelengths, and oscillator strengths for S_n (n = 1–10) states based on TPH molecules

Excited State	Vertical Excitation Energy (eV)	Absorption Wavelength (nm)	Oscillator Strength
S_1	4.0148 eV	308.82 nm	0
S_2	4.2241 eV	293.52 nm	0
S_3	4.4580 eV	278.11 nm	0.0005
S_4	4.4582 eV	278.10 nm	0.0005
S_5	4.8142 eV	257.54 nm	0.359
S_6	4.8143 eV	257.53 nm	0.3591
S_7	5.0915 eV	243.51 nm	0.3875
S_8	5.0916 eV	243.51 nm	0.3878
S_9	5.1945 eV	238.68 nm	0
S_{10}	5.7755 eV	214.67 nm	0.0095

Table S4. Calculated vertical emission energies, emission wavelengths, and oscillator strengths for S_n (n = 1–10) states based on TPH molecules

Excited State	Vertical Emission Energy (eV)	Emission Wavelength (nm)	Oscillator Strength
S_1	3.7463 eV	330.95 nm	0
S_2	3.9636 eV	312.81 nm	0
S_3	4.3280 eV	286.47 nm	0
S_4	4.3280 eV	286.47 nm	0
S_5	4.6197 eV	268.38 nm	0.4185
S_6	4.6197 eV	268.38 nm	0.4186
S_7	4.9481 eV	250.57 nm	0.3821
S_8	4.9481 eV	250.57 nm	0.3823
S_9	5.1741 eV	239.62 nm	0
S_{10}	5.6933 eV	217.77 nm	0.0097

Table S5. Calculated vertical emission energies, emission wavelengths, and oscillator strengths for T_n (n = 1–10) states based on TPH molecules

Excited State	Vertical Emission Energy (eV)	Emission Wavelength (nm)	Oscillator Strength
T_1	2.6465 eV	468.48 nm	0
T_2	3.3569 eV	369.35 nm	0
T_3	3.3570 eV	369.33 nm	0
T_4	3.4928 eV	354.97 nm	0
T_5	3.4928 eV	354.97 nm	0
T_6	3.6356 eV	341.03 nm	0
T_7	4.1983 eV	295.32 nm	0
T_8	4.2373 eV	292.60 nm	0
T_9	4.2374 eV	292.59 nm	0
T_{10}	4.5520 eV	272.37 nm	0

Table S6. Spin-orbit coupling (SOC) calculation results for TPH molecules (cm^{-1})

	S_0	S_1	S_2	S_3	S_4	S_5
T_1	0.23	2.74	0.01	0.07	0.11	0.05
T_2	0.03	0.12	0.05	1.35	0.54	0.26
T_3	0.52	0.07	0.08	1.39	0.17	0.32
T_4	0.37	0.10	0.08	0.28	0.17	0.10
T_5	0.19	0.03	0.05	0.32	0.49	0.6

Table S7. Crystallographic data and structural refinement details for the TPH crystal

**Compound**	TPH crystal at 100 K	TPH crystal at 298 K
Formula	$C_{18}H_{12}$	$C_{18}H_{12}$
Temperature / K	100	298
Formula weight	228.28	228.28
Crystal system	orthorhombic	orthorhombic
Space group	$P2_12_12_1$	$P2_12_12_1$
a / Å	5.2667(3)	5.2752(3)
b / Å	12.8822(7)	13.1795(8)
c / Å	16.6351(9)	16.7642(10)
α / °	90	90
β / °	90	90
γ / °	90	90
Volume / Å^3	1128.64(11)	1165.52(12)
Z	4	4
Density / (g cm^{-3})	1.343	1.332
μ /mm^{-1}	0.076	0.074
F_{000}	480.0	480.0
Reflections collected	17485	16893
Independent reflections	3167	3263
CCDC No.	2389555	2389556

Table S8. Comparison of optical loss coefficients of TPH crystals with previously reported flexible organic crystals.

Name	Optical loss coefficients / dB mm^{-1}	Ref
P-2	0.058	41
S-BPEMP	0.076	33
HDBP	0.125	9
DPPC	0.129	40
Cry-1R	0.139	37
p-DPTPD	0.144	43
compound 1	0.160	31
DPIN	0.270	1
HDED	0.272	32
DBBZL	0.285	19
**TPH**	**0.296**	**This work**
DCA	0.900	54
PTX-2CF_3	82.46	17
Ant-CF_3	136.0	5
BTD2CF_3	159.7	12

References

1. APEX3, v2015.52, Bruker AXS Inc., Madison, WI, **2015**.

2. Dolomanov, O. V.; Bourhis, L. J.; Gildea, R. J.; Howard, J. A. K.; Puschmann, H. OLEX2: a complete structure solution, refinement, and analysis program. *J. Appl. Crystallogr.* **2009**, *42*, 339 – 341. DOI: 10.1107/S0021889808042726

3. Spek, A. L. Single-crystal structure validation with the program PLATON. *J. Appl. Crystallogr.* **2003**, *36*, 7 – 13. DOI: 10.1107/S0021889802022112

4. Macrae, C. F. et al. Mercury CSD 2.0-New features for the visualization and investigation of crystal structures. *J. Appl. Crystallogr.* **2008**, *41*, 466–470. DOI: 10.1107/S0021889807067908

Legends for the Supplementary Movies

Movie S1. Change in the phosphorescence emission from a TPH crystal while it is being bent (the initial part of the movie shows the excitation by UV light).

Movie S2. Waveguiding of the delayed phosphorescence through a straight TPH crystal (the initial part of the movie shows the excitation by UV light).

Movie S3. Temperature dependence of the waveguiding capability. One end of the crystal is at 298 K and the other one at 77 K. When the cold end is excited (365 nm), the light is transduced to the warm end. When the ware end was excited, no light was transduced to the cold end. The video is shown at 5-times the actual speed.

Movie S4. Spatial control over the emission output. By exciting one end of the crystal with a 365 nm UV light, the position of the output light signal from the other end changes as the crystal is mechanically bent. The video is shown 4-times the actual speed.

Movie S5. Spatial control over multiple light output. The crystal is excited at one end kept at 77 K, and mechanical bent, whereupon it transduces light to its other end, which is at 298 K. The process is repeated multiple times. The video is shown 3-times the actual speed.

Movie S6. Spatial control over the light output. The crystal is excited at one end kept at 77 K, and mechanical bent, whereupon it transduces light to its other end, which is at 298 K.

Movie S7. Phosphorescence waveguiding of TPH crystals in a biological tissue. One end of the crystal was excited with UV light at 77 K, and then the UV light was turned off. The light was transduced to the other end, which was at 298 K.
